# Divergence Across Niche Dimensions Reveals Species' Ecological Roles

**DOI:** 10.1111/ele.70173

**Published:** 2025-07-11

**Authors:** Marcelo Magioli, Vinicius Alberici, Elildo A. R. Carvalho, Nina Attias, Katia Maria Paschoaletto Micchi de Barros Ferraz, Marcelo Zacharias Moreira, Arnaud L. J. Desbiez, Adriano Garcia Chiarello

**Affiliations:** ^1^ Laboratório de Ecologia e Conservação (LAEC), Departamento de Biologia, Faculdade de Filosofia, Ciências e Letras Universidade de São Paulo (USP) Ribeirão Preto São Paulo Brazil; ^2^ Instituto Pró‐Carnívoros Atibaia São Paulo Brazil; ^3^ Centro Nacional de Pesquisa e Conservação de Mamíferos Carnívoros (CENAP) Instituto Chico Mendes de Conservação da Biodiversidade (ICMBio) Atibaia São Paulo Brazil; ^4^ Instituto de Conservação de Animais Silvestres (ICAS) Campo Grande Mato Grosso do Sul Brazil; ^5^ Laboratório de Ecologia, Manejo e Conservação de Fauna Silvestre (LEMaC), Departamento de Ciências Florestais Escola Superior de Agricultura “Luiz de Queiroz” (ESALQ), Universidade de São Paulo (USP) Piracicaba São Paulo Brazil; ^6^ Laboratório de Ecologia Isotópica, Centro de Energia Nuclear na Agricultura Universidade de São Paulo Piracicaba São Paulo Brazil; ^7^ Instituto de Pesquisas Ecológicas, Organização Não Governamental Nazaré Paulista São Paulo Brazil; ^8^ Royal Zoological Society of Scotland (RZSS) Edinburgh UK

**Keywords:** activity pattern, camera trapping, ecological niche, habitat use, natural history, occupancy models, resource use, roadkills, stable isotopes, Xenarthra

## Abstract

Natural history data are essential for understanding species' ecological roles, supporting applied research and guiding conservation efforts. However, significant gaps in ecological knowledge limit our understanding, requiring complementary approaches to bridge them. Using an integrative analytical framework, we explored multiple niche dimensions of poorly known co‐occurring xenarthran insectivores, uncovering shared and unique roles within this guild. Our findings revealed divergences among most species' pairs across three niche dimensions while emphasising distinct ecological roles within a three‐tier trophic structure. Habitat use was strongly influenced by resource availability, with species exploiting both natural and anthropogenic habitats, reflecting a double‐edged trade‐off. Spatial and trophic patterns mirrored each other, demonstrating the interconnectedness of diet and habitat use, with activity patterns further aligning with these trends. These findings challenge assumptions of ecological redundancy and highlight the complexity of guild‐level interactions, emphasising critical knowledge gaps in biodiversity and its essential contributions to global ecosystem processes.

## Graphical abstract

Natural history gaps hinder our understanding of species' ecological roles and their contributions to ecosystem processes. Using an integrative framework, we analysed multiple niche dimensions of co‐occurring xenarthran insectivores, revealing divergent trophic, spatial and temporal patterns that reflect distinct ecological roles. Our results challenge assumptions of ecological redundancy and underscore the importance of natural history data for understanding biodiversity and ecosystem functioning.

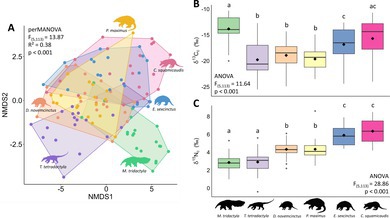

## 1 Introduction

Determining species' roles in ecosystems is a major goal in ecology. While it is relatively straightforward to infer the ecological role of well‐studied organisms based on phylogeny or morphology, this is challenging for poorly studied ones, especially in the tropics, where biodiversity is exceptionally high (Gaston 2000). Ecological information on tropical organisms is often limited (Oliveira et al. 2016) due to the scarcity of studies and most species' rarity, elusiveness and cryptic behaviour. These knowledge gaps are particularly pronounced for taxonomic groups that are difficult to capture or monitor, such as the Xenarthra (Superina and Loughry 2015), a superorder comprising 42 extant species across the orders Cingulata and Pilosa, including sloths, anteaters and armadillos (Mammal Diversity Database 2025). These species are primarily confined to the Neotropics (Emmons and Feer 1997), and most are poorly studied regarding their biology, natural history and ecological and functional roles (Superina and Loughry 2015). For example, little is known about their habitat preferences and responses to habitat modification and expanding agriculture.

Most biodiversity assessments assume that closely related species fulfil similar or redundant roles and group them into broad categories (e.g., trophic guilds and functional groups) (Gorczynski et al. 2021), but this approach can be misleading as it may under‐ or overestimate organisms' importance for ecosystem functioning. In this context, bridging knowledge gaps in species' natural history is essential for understanding their ecological roles. Since natural history data can reveal divergences in ecological niches (Nosil 2012), examining multiple niche dimensions allows connecting patterns of resource use, habitat preferences and activity patterns (MacArthur and Levins 1967), and provides a basis for testing ecological theories, such as the Niche Variation Hypothesis (Van Valen 1965). However, acquiring data to support the simultaneous analysis of multiple niche dimensions is daunting. To address this, employing multifaceted analytical frameworks is crucial, as they integrate ecological theory with modern sampling techniques and analytical tools (e.g., Ascanio et al. 2024). These complementary approaches enhance traditional methods and deepen our understanding of species' ecological responses across broad spatial and temporal scales.

For instance, stable isotope analysis is a powerful tool for uncovering species' resource and habitat use and disentangling trophic relationships among organisms (Magioli et al. 2019). Its explanatory power is further enhanced when combined with camera trapping, which is especially effective for sampling elusive and cryptic species (Tobler et al. 2008). Integrating such complementary approaches is essential as they provide valuable biological models for testing hypotheses, investigating niche divergences and understanding species‐specific responses to environmental changes.

Here, we employed an integrative analytical framework to explore the differences and similarities within a guild of co‐occurring xenarthran insectivores (anteaters and armadillos) across the trophic, spatial and temporal dimensions of their niches. By examining divergences across these dimensions, we provide species‐ and population‐level insights that clarify their shared and unique ecological roles within the ecosystem, and evaluate how these differences may influence biodiversity assessments and inform effective conservation strategies.

Our main questions (Q) and corresponding hypotheses (H) are as follows:

Do closely related species differ in the use of food resources?

*Xenarthrans that share morphological traits* (*e.g., body size and locomotor habits) and behavioural characteristics* (*e.g., foraging substrate*) (*Emmons and Feer* 1997) *and are evolutionarily close* (*Gibb* et al. 2016) *will exhibit similar resource use. These similarities will be reflected by their isotopic values and niches, the origin of food resources* (*e.g., open areas, forests and agricultural areas*) *and diet composition*.

Do agricultural areas benefit armadillos and anteaters?

*According to the Niche Variation Hypothesis* (*Van Valen* 1965), *when food resources become limited, as in human‐modified landscapes, species may resort to non‐preferred items* (*Marshall and Wrangham* 2007) *or exploit resources from agricultural areas* (*e.g., Magioli* et al. 2019; *Manlick and Pauli* 2020). *Therefore, species are likely to benefit from agricultural areas in these landscapes, both as a food source and as part of their habitat*.

Are there substantial differences in the trophic structure of an insectivore guild?

*Because armadillos and anteaters are primarily insectivores* (*i.e., consumers of animal matter*), *they are expected to exhibit elevated nitrogen isotopic values* (*e.g., Codron* et al. 2007; *Kelly* 2000). *Although intra‐ and inter‐specific variation in nitrogen isotopic values is anticipated, species within this guild will occupy the same trophic level with similar average values* (*Post* 2002).

Does land cover explain resource use?

*As carbon isotopes drastically differ between C*_*3*_ *and C*_*4*_ *plants* (*Tieszen and Boutton* 1989), *species primarily associated with forests (dominated by C*_*3*_ *plants) will exhibit lower carbon isotopic values than those using mixed habitats* (*e.g., Magioli* et al. 2022, 2023), *which include forests, open vegetation and agricultural areas* (*containing both C*_*3*_ *and C*_*4*_ *plants*). *Furthermore, species frequently using agricultural areas* (*dominated by C*_*4*_ *plants*) *will display the highest carbon isotopic values. This pattern will also align with the species' habitat use within the landscape*.

## 2 Methods

### 2.1 Study Areas

The study was conducted in two landscapes within the Cerrado and Pantanal biomes of Mato Grosso do Sul, Brazil (Figure S1A). The Cerrado landscape, situated in the centre of the state, encompasses three paved roads where sampling occurred: BR‐262, BR‐267 and MS‐040 (Figure S1B,C). The landscape within a 10‐km buffer around these roads is predominantly composed of agriculture and pasture (70%), while natural forests and grasslands cover the remainder (Projeto MapBiomas 2023). The Pantanal landscape, located in the Nhecolândia subregion, features a diverse range of largely pristine habitats, including permanent and temporary ponds, natural and exotic open grasslands, open and closed savannas and semi‐deciduous forests (Projeto MapBiomas 2023) (Figure S1D).

### 2.2 Hair Sample Collection

In the Cerrado landscape, we collected hair samples from 20 roadkilled individuals for each of five xenarthran species: *Myrmecophaga tridactyla* (giant anteater), *Tamandua tetradactyla* (southern anteater), *Euphractus sexcinctus* (six‐banded armadillo), *Dasypus novemcinctus* (nine‐banded armadillo) and *Cabassous squamicaudis* (southern naked‐tailed armadillo). Samples were collected during roadkill surveys along the three highways from January 2017 to February 2019, resulting in a sampling effort of 85,486 km. From the Pantanal landscape, we included 19 samples from captured *Priodontes maximus* (giant armadillo) (Magioli et al. 2023). In total, we analysed hair samples from 119 individuals across six species. Although samples are from different biomes, carbon isotopic values from the vegetation are similar [Cerrado:−28.9‰; Pantanal:−30.1‰; (Martinelli et al. 2021)]. Additionally, the landscape composition regarding the proportions of C_3_ and C_4_ plants is comparable (Figure S1), which allows for a meaningful comparison among species (details on the studied species and hair collection are provided in Appendix S1). Species nomenclature followed Abreu et al. (2024).

### 2.3 Camera Trapping

In the Cerrado landscape, we deployed camera traps within 10‐km buffer zones surrounding BR‐267 and MS‐040 roads (Figure S3). We randomly placed non‐overlapping circular units within the buffer zones to determine sampling sites, designating 60 sites in each area, totalling 120 sampling sites. We installed camera traps on tree trunks at knee height, operating continuously for approximately 30 days. Sampling occurred during the dry season (April to September), with data collection in MS‐040 in 2018 and BR‐267 in 2019, totalling 10,529 trap‐days, which detected all six species (details in Appendix S1).

### 2.4 Stable Isotope Analysis

Hair samples were cleaned with 70% alcohol to remove residues, dried with absorbent paper, cut into small pieces and stored in thin capsules. We used a CHN‐1110 Elemental Analyzer (Carlo Erba, Milan, Italy) to combust the samples and separate the resulting gases in a chromatographic column. The gases were analysed with a coupled continuous flow isotope ratio mass spectrometer (Delta Plus, Thermo Scientific, Bremen, Germany) to determine the isotopic composition. The carbon and nitrogen isotopic values were expressed in delta notation (δ^13^C, δ^15^N) in parts per mil (‰) relative to the V‐PDB (Vienna‐Pee Dee Belemnite) and atmospheric N_2_ standards, respectively. Delta values were calculated using the following equation δX = [(R_sample_/R_standard_)−1] multiplied by 1000, where X represents the stable carbon (^13^C) or nitrogen isotopes (^15^N), and R is the isotope ratio (^13^C/^12^C or ^15^N/^14^N). We replicated individual material for only 10% of the samples. However, the precision of the analytical method was validated with 22 replicates of an internal standard across all batches, showing a precision of 0.1‰ for both carbon and nitrogen. The samples were anchored to international standards using reference materials: NBS‐19 and NBS‐22 for carbon, and IAEA‐N1 and IAEA‐N2 for nitrogen (details on how to interpret isotopic values are provided in Appendix S1).

### 2.5 Landscape Metrics

We assessed landscape composition representing the availability of C_3_ or C_4_ resources across the study areas and their potential influence on species resource and habitat use at different spatial scales. We utilised the MapBiomas land cover map (Projeto MapBiomas 2023) and calculated the variables using the package *landscapemetrics_v2.2.1* (Hesselbarth et al. 2019) available in R 4.3.2 (R Core Team 2024). Five variables were selected for analysis: semideciduous forests and savanna (sources of C_3_ carbon), pasture and grasslands (sources of C_4_ carbon) and mosaic of uses (a mixed source of both C_3_ and C_4_ carbon). We calculated the percentage of each land cover at four scales: the species home range (Table S1), and within buffers of 500, 1000 and 2000 m. Evaluating different spatial scales allows for discussing effects at the individual (home range) and population (larger scales) levels. These variables were used as explanatory factors in linear regression models with isotopic values, where roadkill sites served as reference points, and as covariates in occupancy models, using camera trap location as reference sites (details of the variables' calculation and species' home range are provided in Appendix S2).

### 2.6 Data Analysis

We performed all analyses in R 4.3.2 and used the *ggplot2_v3.5.2* package (Wickham 2016) for graphical implementation. The full workflow is presented in Figure S4.

### 2.7 Trophic Dimension

#### 2.7.1 Resource Use

We corrected all δ^13^C and δ^15^N values (henceforth, δ^13^C_c_ and δ^15^N_c_) for the subsequent analyses using trophic discrimination factors (Δ^13^C and Δ^15^N, respectively) estimated by the *SIDER_v1.0.0.0* package (Healy et al. 2018) (Table S2; details in Appendix S3). To assess differences in isotopic values among species, we first examined the multivariate homogeneity of group dispersions using 999 permutations. Next, we conducted a Permutational Multivariate Analysis of Variance (perMANOVA) with 999 permutations to visualise the multivariate patterns through a Nonmetric Multidimensional Scaling (NMDS) based on a Euclidean dissimilarity matrix. We then evaluated the differences between species pairs. Analyses were conducted using the *vegan_v2.6–8* (Oksanen et al. 2024) and *pairwiseAdonis_v0.0.1* packages (Arbizu 2017). In addition, we compared isotopic values among species using ANOVA and Tukey tests. Homogeneity and residuals' normality were tested with Levene and Shapiro–Wilk tests, respectively.

To determine the origin of food items consumed by insectivorous Xenarthra, we adapted the analytical approach described by Magioli et al. (2014). This method calculates the proportion of C_3_/C_4_ carbon in each sample, enabling their classification into three groups: C_3_‐based (> 70% C_3_ carbon), C_4_‐based (< 30% C_3_ carbon), and mixed diets (30%–70% C_3_ carbon). We compared the proportions of individuals per species and across all species assigned to each dietary group (details in Appendix S3). To further assess resource use similarities among species, we calculated the size and overlap of isotopic niches using a kernel utilisation density estimator, as implemented in the *rKIN_v1.0.4* package (Eckrich et al. 2020). This analysis utilised the 50% contour to represent core resource use and the 95% contour to encompass the full range of dietary niches.

#### 2.7.2 Feeding Habits

We conducted a comprehensive literature search on the species' feeding habits in Web of Science, Scopus and Google Scholar from January to April 2024, compiling 29 studies for the six species (Table S3). For studies with more than five samples, we quantified the contribution of invertebrates (highlighting ants, termites and coleopterans), vertebrates and plant material to each species' diet. Based on the frequency of occurrence of these food items, we classified the species as strictly myrmecophagous or insectivorous‐omnivorous (Table S4). We presented the contribution of the main food items in each species' diet by averaging their frequency of occurrence across selected studies and using radar plots (details in Appendix S3).

### 2.8 Spatial Dimension

#### 2.8.1 Resource Use

We performed linear regression models to examine whether landscape composition (forest, savanna, grassland, pasture and mosaic of uses) influences the resource use measured by isotopic values (δ^13^C_c_ or δ^15^N_c_) of insectivorous xenarthrans at four scales (home range, 500, 1000 and 2000 m). To select the most relevant variables at the optimal scale of influence for each species, we employed a two‐step Bayesian variable selection procedure (Tenan et al. 2014). First, we constructed a model including all explanatory variables with isotopic values as the dependent variables, with a Bernoulli distributed indicator for each explanatory variable to associate it with an inclusion probability value. We then selected the four highest‐ranked variables identified by the indicator, excluded those with correlations greater than 0.5, and adjusted the final models with the remaining variables (Table S5). Model assumptions were verified with the *performance_v0.14.0* package (Lüdecke et al. 2021) (details in Appendix S3).

#### 2.8.2 Habitat Use

We employed Bayesian single‐species occupancy models to assess the effects of landscape composition on each species' detection and occupancy probabilities (MacKenzie et al. 2002). We interpreted occupancy probability as habitat use, that is, the probability that a given sampling site was used by the species during the sampling period. To select the best variables at the optimal scale of influence for each species, we employed the same two‐step Bayesian variable selection procedure described above (Tenan et al. 2014) (Table S6). We fitted the models in JAGS_v4.3.2 (Plummer 2023) using the *R2jags_v0.8–5* package (Su and Yajima 2024). We considered a predictor effect supported when the parameters' 95% posterior credible interval did not include zero (details in Appendix S3).

### 2.9 Temporal Dimension

#### 2.9.1 Activity Patterns

Using camera trap data, we characterised the species' activity patterns as diurnal, nocturnal or cathemeral (active during day and night) and assessed the overlap between pairs. We used species' independent records and conducted the analyses using the *activity_v1.3.4* (Rowcliffe 2023) and *overlap_v0.3.9* (Meredith et al. 2024) packages.

## 3 Results

### 3.1 Trophic Dimension

#### 3.1.1 Resource Use

**FIGURE 1:**
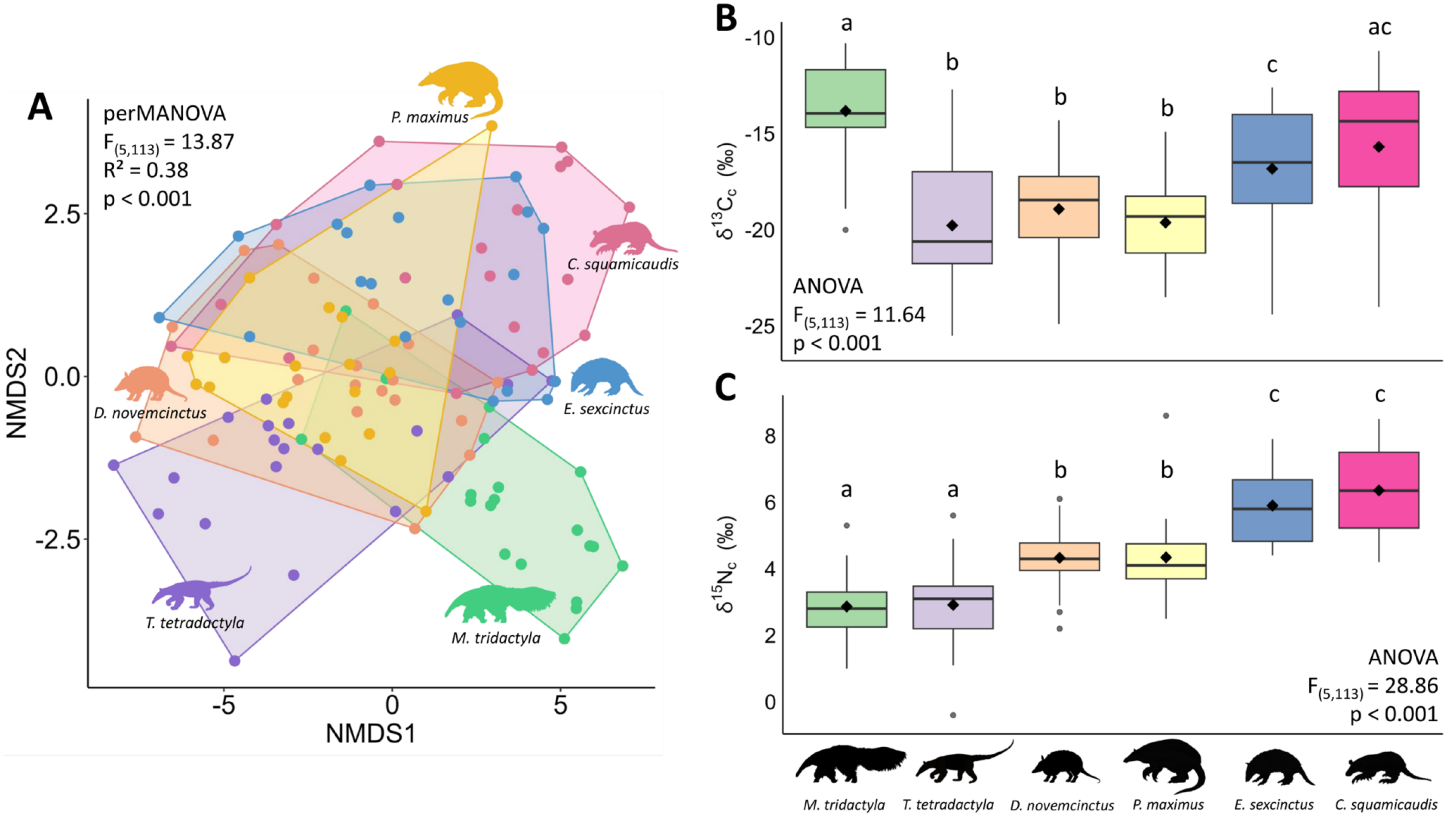
(A) Multivariate patterns of isotopic values (δ^13^C_c_ and δ^15^N_c_) among anteaters and armadillos, illustrated by Nonmetric Multidimensional Scaling (NMDS). Comparison of δ^13^C_c_ (B) and δ^15^N_c_ values (C) for anteaters and armadillos in the Cerrado and Pantanal landscapes, Mato Grosso do Sul, Brazil. Boxplots display means (diamonds), medians, quartiles and outliers. Small case letters indicate significant differences among species (carbon, *p* ≤ 0.05; nitrogen, *p* ≤ 0.005).

**FIGURE 2:**
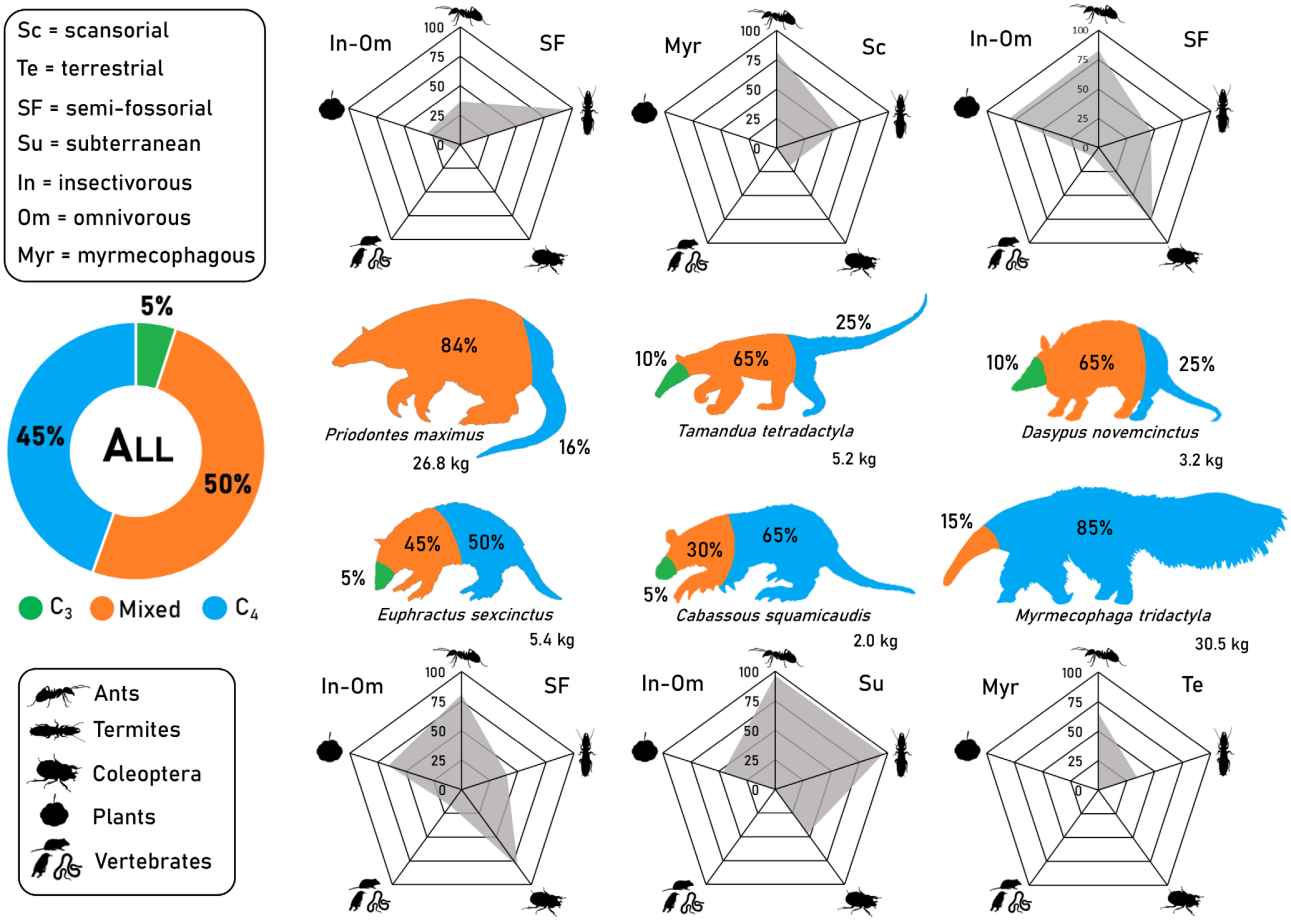
Silhouettes display the percentage of anteater and armadillo individuals with C_3_, mixed, and C_4_ diets in the Cerrado and Pantanal landscapes, Mato Grosso do Sul, Brazil, and for all individuals combined (donut plot), alongside their mean body mass. Radar plots illustrate the average frequency of occurrence of key food items in each species' diet, their dietary classification and locomotor habits.

Anteaters and armadillos presented a wide variation in δ^13^C_c_ (from −25.5‰ to −10.3‰) and δ^15^N_c_ values (from −0.4‰ to 8.6‰) (Table S7). We observed a homogeneous multivariate dispersion of isotopic values among species (*F*_(5,113)_ = 1.07, *p* = 0.40), with significant differences among them (perMANOVA, *F*_(5,113)_ = 13.87, *R* ^2^ = 0.38, *p* = 0.001) (Figure 1A), as exhibited by 10 out of 15 species pairs (Table S8). Among the five pairs without significant differences, only one—*C. squamicaudis* and *E. sexcinctus* —involved closely related species, both from the family Chlamyphoridae. The δ^13^C_c_ values revealed substantial intraspecific variation in foraging patterns, spanning from strictly C_3_ to strictly C_4_ diets. Species primarily foraging in open habitats dominated by C_4_ carbon ( *M. tridactyla* , *E. sexcinctus* and *C. squamicaudis*) contrasted with those incorporating substantial amounts of C_3_ carbon, predominantly found in forested habitats ( *T. tetradactyla* , *D. novemcinctus* and *P. maximus* ) (ANOVA, *F*_(5,113)_ = 11.64, *p* < 0.001, Figure 1B). This pattern becomes more evident when considering the composition of each dietary group: most individuals used a mixture of C_3_ and C_4_ resources (50%), followed closely by those with strict C_4_ diets (45%), and only a few individuals with strict C_3_ diets (5%) but belonging to four different species (Figure 2). Although δ^15^N_c_ values showed significant intraspecific variation, we observed a clear trophic structure across three distinct levels (ANOVA, *F*_(5,113)_ = 28.86, *p* < 0.001) (Figure 1C). Model assumptions evaluation is presented in Appendix S4.

**FIGURE 3:**
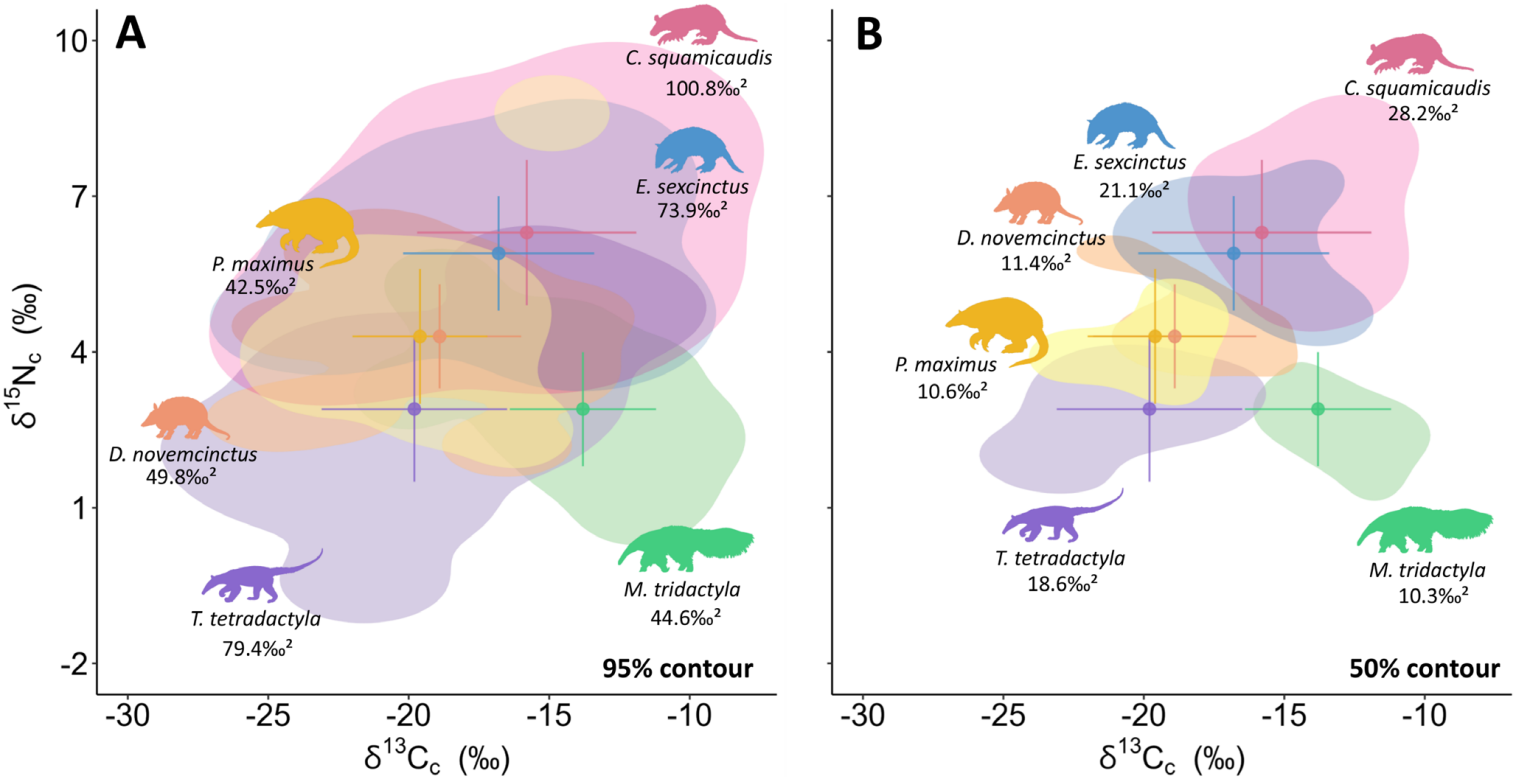
Isotopic niches of anteater and armadillo species in the Cerrado and Pantanal biomes, Mato Grosso do Sul, Brazil, are depicted with 95% (A) and 50% (B) contour areas. The values below the silhouettes represent the isotopic niche size for each contour (‰^2^).

Considering the full dietary niche (95% contour), most species pairs (62.5%) exhibited overlaps below 50% (Figure 3A, Table S9). *Cabassous squamicaudis* showed the largest isotopic niche and the highest overlaps, highlighting its high intraspecific variation and broad feeding preferences. In contrast, *M. tridactyla* and *P. maximus* exhibited the smallest niches and the lowest overlaps, suggesting more specialised diets. The differentiation in resource use was strengthened by the generally low overlaps (< 10%) among species within the core dietary niche (50% contour) (Figure 3B, Table S10), with notable exceptions of higher overlap between *D. novemcinctus* and *P. maximus* (50.5% and 46.6%), and between *C. squamicaudis* and *E. sexcinctus* (65.6% and 49.1%).

#### 3.1.2 Feeding Habits

Invertebrates were present in all samples across species in the compiled studies (Table S3). Anteaters were strictly myrmecophagous, while armadillos consumed a diverse range of invertebrates, plant material and small vertebrates, classifying them as insectivorous‐omnivorous. Diet composition mirrored isotopic niche sizes for most species (Figures 2 and 3; Table S4).

### 3.2 Spatial Dimension

#### 3.2.1 Resource Use

**FIGURE 4:**
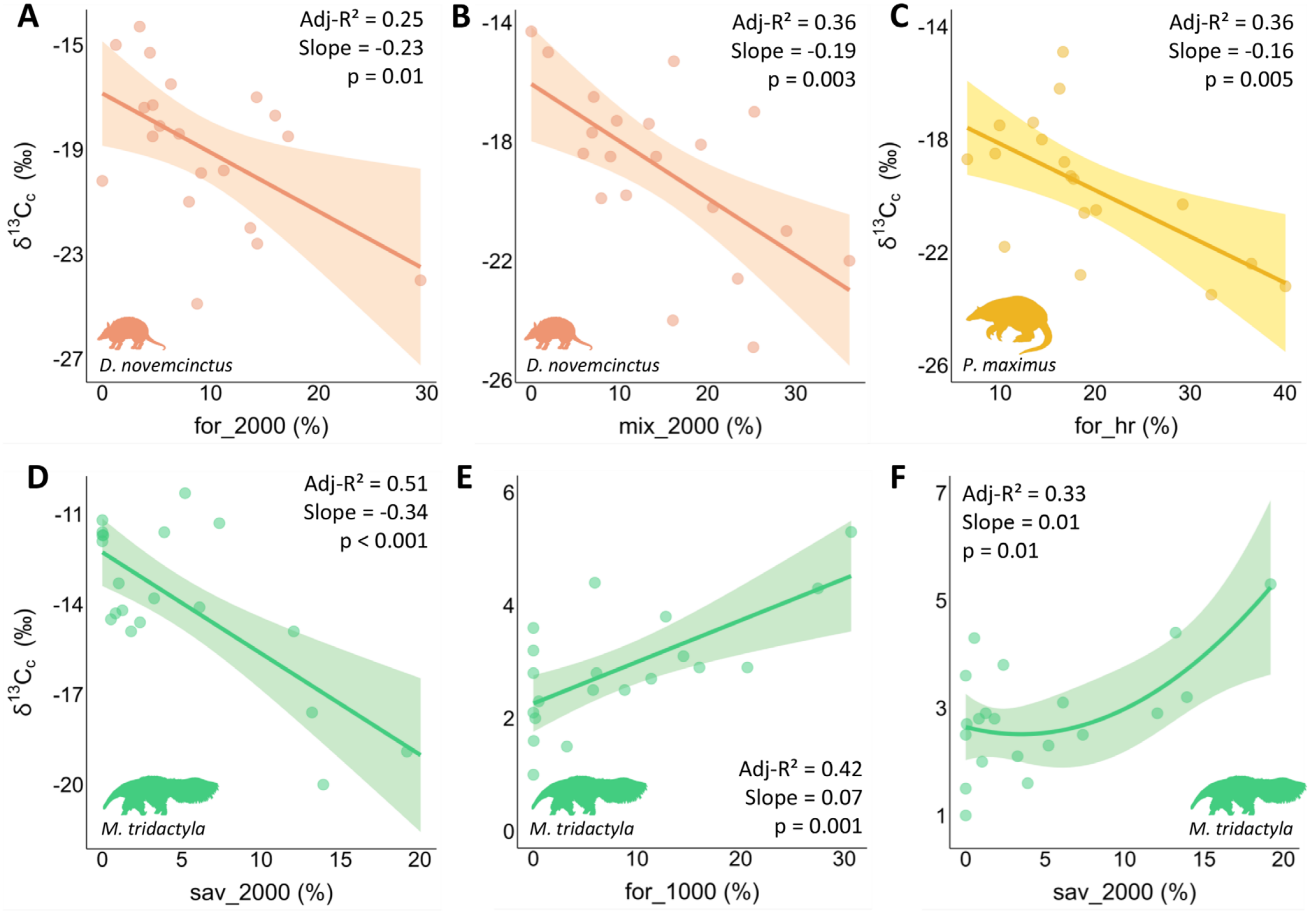
Relationships between isotopic values (δ^13^C_c_ or δ^15^N_c_) and landscape composition variables at different scales of effect for anteaters and armadillos in the Cerrado and Pantanal landscapes, Mato Grosso do Sul, Brazil. (A, B) *Dasypus novemcinctus* ; (C) *Priodontes maximus* ; (D, E, F) *Myrmecophaga tridactyla* . Habitat categories: Forest (for), savanna (sav) and mosaic of uses (mix). The numbers following the underscore represent the scale of effect (in m); hr, home range.

Three species showed significant changes in isotopic values as landscape composition shifted at different scales, mainly related to forest formations (C_3_ carbon) (Figure 4; Table S11). As forest cover increased, *D. novemcinctus* (Adj‐*R* ^2^ = 0.25, slope = −0.23, *p* = 0.01; Figure 4A) and *P. maximus* (Adj‐*R* ^2^ = 0.36, slope = −0.16, *p* = 0.005; Figure 4C) exhibited a decrease in δ^13^C_c_ values at the population and individual levels, respectively. *Dasypus novemcinctus* exhibited a similar pattern with mixed cover at the population level (Adj‐*R* ^2^ = 0.36, slope = −0.19, *p* = 0.003; Figure 4B). Although *M. tridactyla* presented the highest proportion of C_4_ carbon in its diet, its δ^13^C_c_ values decreased with increasing savanna cover at the individual level (Adj‐*R* ^2^ = 0.51, slope = −0.34, *p* < 0.001; Figure 4D). *Myrmecophaga tridactyla* was the only species that exhibited responses in stable nitrogen isotopes to landscape composition, with δ^15^N_c_ values increasing as forest (Adj‐*R* ^2^ = 0.42, slope = 0.07, *p* = 0.001; Figure 4E) and savanna cover increased at the individual level (Adj‐*R* ^2^ = 0.33, slope = 0.01, *p* = 0.01; Figure 4F). Model assumptions evaluation is presented in Appendix S4.

#### 3.2.2 Habitat Use

**FIGURE 5:**
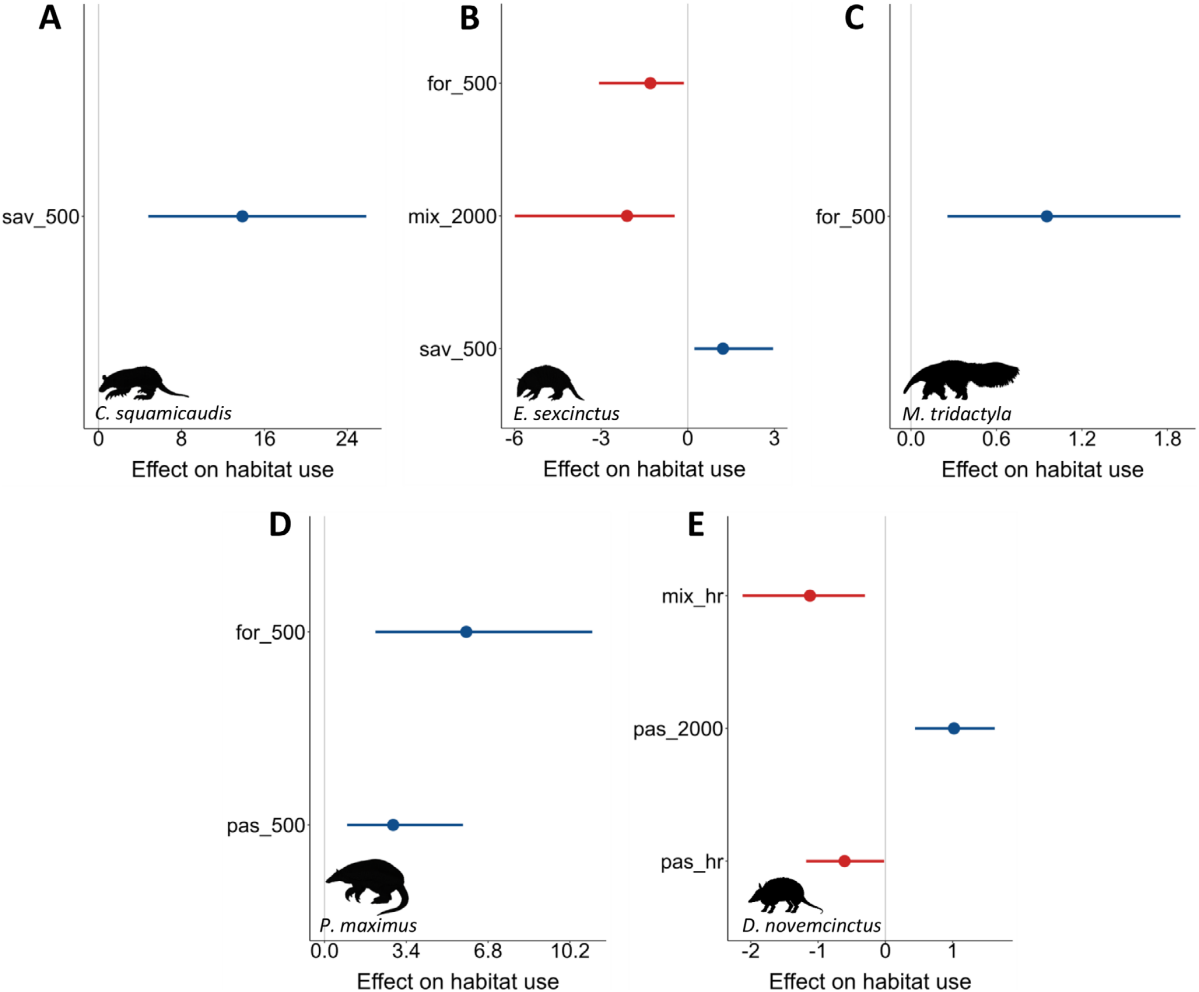
Results of the Bayesian single‐species occupancy model, showing the magnitude and direction (Bayesian means ±95% credible intervals) of the posterior distributions of landscape composition covariates on the habitat use of anteaters and armadillos in the Cerrado landscape, Mato Grosso do Sul, Brazil. (A) *Cabassous squamicaudis*; (B) *Euphractus sexcinctus* ; (C) *Myrmecophaga tridactyla* ; (D) *Priodontes maximus* ; (E) *Dasypus novemcinctus* . Blue and red lines denote positive and negative effects, respectively. Habitat categories: Forest (for), savanna (sav), pasture (pas) and mosaic of uses (mix). The numbers following the underscore represent the scale of effect (in m); hr, home range.

The habitat use of *C. squamicaudis* and *E. sexcinctus* was positively related to savannas (Figure 5A,B), while *M. tridactyla* and *P. maximus* were positively influenced by forests (Figure 5C,D), all at the individual level. Surprisingly, *P. maximus* and *D. novemcinctus* habitat use was positively related to pastures at different scales (Figure 5D,E). Conversely, mixed cover negatively impacted the habitat use of *E. sexcinctus* and *D. novemcinctus* at the individual level, with *E. sexcinctus* also being negatively affected by forests and *D. novemcinctus* by pastures at the individual level (Figure 5B,E).

### 3.3 Temporal Dimension

#### 3.3.1 Activity Patterns

**FIGURE 6:**
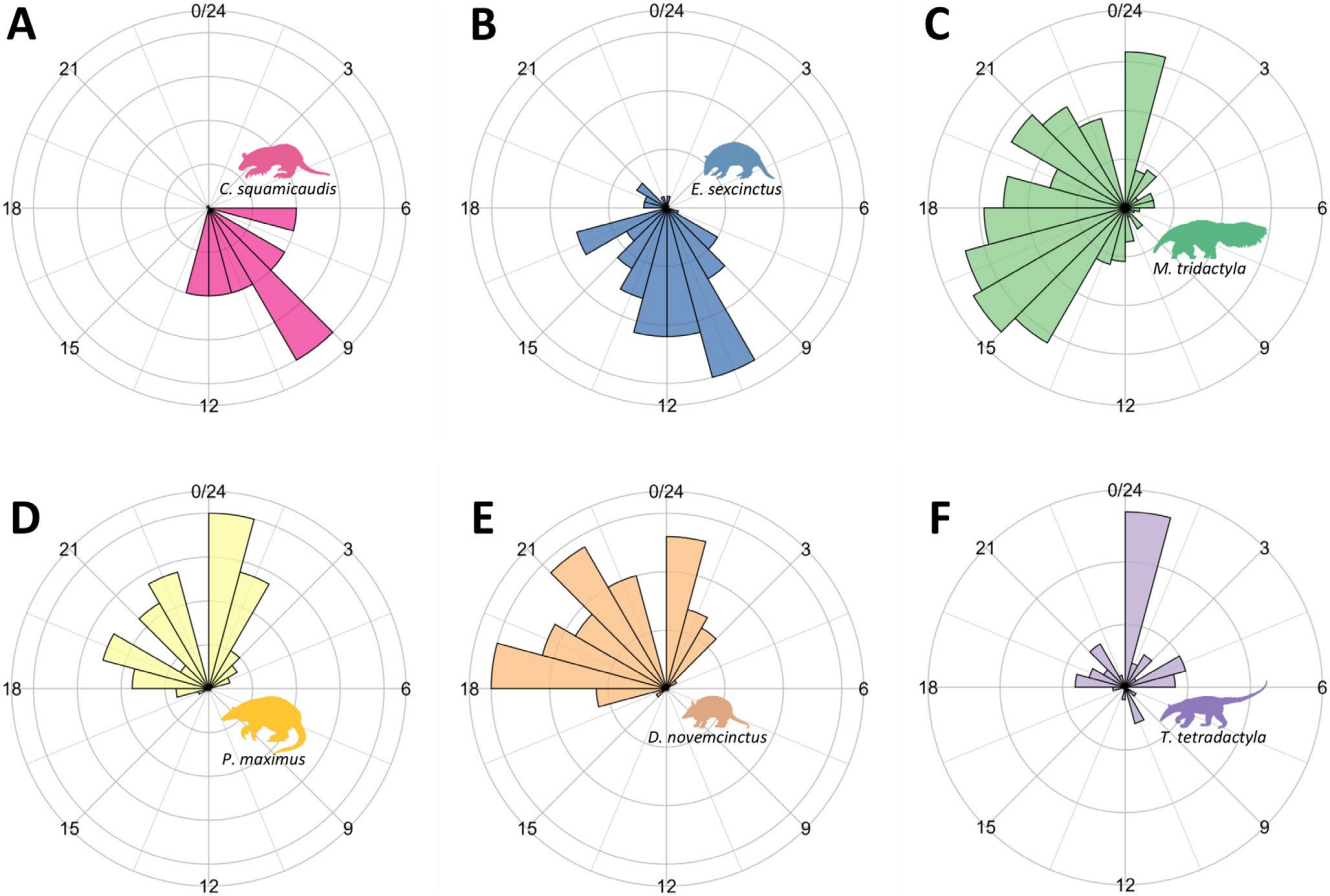
Activity periods for anteaters and armadillos in the Cerrado landscape, Mato Grosso do Sul, Brazil. (A) *Cabassous squamicaudis*; (B) *Euphractus sexcinctus* ; (C) *Myrmecophaga tridactyla* ; (D) *Priodontes maximus* ; (E) *Dasypus novemcinctus* ; (F) *Tamandua tetradactyla* .

Species converging on the trophic and spatial dimensions exhibited similar activity patterns. For example, *C. squamicaudis* and *E. sexcinctus* were predominantly diurnal (Figure 6A,B), while *P. maximus* and *D. novemcinctus* were nocturnal (Figure 6D,E), with high overlaps (0.63 and 0.83, respectively) (Table S12). *Myrmecophaga tridactyla* was the only cathemeral species (Figure 6C), a pattern consistent with its distinctive resource use.

## 4 Discussion

### 4.1 Trophic Dimension

#### 4.1.1 Resource Use

The joint analysis of isotopic values, resource origin and diet composition uncovered divergences in xenarthrans' use of food resources, challenging assumptions of ecological redundancy. Divergence in the isotopic bidimensional space was observed in 10 out of 15 species pairs (Table S8), refuting our first hypothesis (H1) and indicating that resource use was not constrained by morphological or phylogenetic similarities. Only one species pair—*C. squamicaudis* and *E. sexcinctus* (both chlamyphorids)—aligned with H1. These species exhibited strong convergence in resource use, sharing similar diet composition, resource origin and trophic position, with large isotopic niches and high core niche overlaps. In contrast, the myrmecophagids *M. tridactyla* and *T. tridactyla*, despite their close phylogenetic relationship (Gibb et al. 2016) and similarities in morphology (Emmons and Feer 1997), diet composition and trophic position, exhibited opposing isotopic niches and negligible core niche overlaps. This contradiction of H1 underscores their complementary ecological roles and suggests distinct resource use strategies despite shared ecological traits.

A striking example of unexpected convergence occurred between two taxonomically distinct species— *D. novemcinctus* and *P. maximus* . Despite belonging to different families (Dasypodidae and Chlamyphoridae, respectively) and exhibiting substantial differences in body mass (tenfold) and home range (125‐times), as well as distinct diet composition and morphology (McDonough and Loughry 2008), these species fully aligned in their trophic position and resource origin. They also shared small isotopic niches and exhibited high core niche overlaps, further contradicting H1. These findings support Schoener's (1974) suggestion that both inherent (e.g., morphology and behaviour) and environmental (e.g., habitat structure and resource availability) factors shape resource partitioning among species. Interpreting niche divergence from a single viewpoint can be misleading (Ascanio et al. 2024), as it can obscure underlying species traits and lead to inaccurate conclusions about their ecological similarities. Such limitations are particularly relevant in applied ecological research, where comprehensive, multi‐dimensional approaches are critical for accurately assessing species' ecological roles and interactions.

#### 4.1.2 The Influence of Agricultural Areas

Xenarthrans strongly relied on C_4_ resources, with 95% of all individuals incorporating them to some extent, emphasising the critical role of agricultural areas as food sources. Given that agricultural landscapes dominate tropical ecosystems globally (Gibbs et al. 2010) while retaining limited native habitats, as seen in the Cerrado, these findings confirm our second hypothesis (H2). They demonstrate that agricultural areas integrate xenarthrans' habitat and align with the observed shift toward alternative food sources when preferred native resources are scarce (Marshall and Wrangham 2007). Similar dietary shifts have been documented in other mammals (Muñoz‐Lazo et al. 2019; Mychajliw et al. 2022), birds (Ferger et al. 2013) and reptiles (Renoirt et al. 2021).

However, human‐modified landscapes present a double‐edged trade‐off: while they offer food resources and habitat opportunities (Magioli et al. 2019; Manlick and Pauli 2020), they also increase species' exposure to threats, highlighting the fragile state of most tropical ecosystems worldwide. For instance, hair samples from the Cerrado were obtained from roadkilled animals, with four of the six species analysed ( *E. sexcinctus* , *D. novemcinctus* , *M. tridactyla* and *T. tetradactyla* ) ranking among the most frequently roadkilled vertebrates in the region (Ascensão et al. 2021). Additionally, chemical compounds and heavy metals from pesticides and fertilisers can bioaccumulate through direct exposure, runoff into natural areas or by the direct consumption of crops, potentially impairing organisms' health and fitness (Jarvis et al. 2013; Medici et al. 2021). Such cumulative risks emphasise the need for conservation strategies that balance the dual role of agricultural landscapes as both habitat and hazard for species.

#### 4.1.3 Trophic Structure

Mammalian insectivores, like carnivores, are expected to exhibit elevated δ^15^N values due to their high reliance on animal matter (e.g., Codron et al. 2007; Kelly 2000). However, our study revealed substantial intra‐ and inter‐specific variation in xenarthrans' δ^15^N_c_ values, spanning a range of 9.0‰. This range corresponds to individuals occupying three distinct trophic levels, assuming stepwise increases of 3‰ (see Post 2002) from the Cerrado vegetation baseline (−0.3‰) (Martinelli et al. 2021), contradicting our third hypothesis (H3), which anticipated minimal differentiation among primarily insectivorous species. This three‐level trophic structure offers deeper insights into species' dietary composition, revealing significant differences and previously undetected similarities. For instance, at the base of this intraguild structure, anteaters exhibited low mean δ^15^N_c_ values (2.9‰), characteristic of primary consumers. This likely reflects their specialised diet of herbivorous ants and termites.

In contrast, armadillos occupied two higher trophic levels, reflecting the consumption of a range of ants and termites (e.g., omnivorous and carnivorous). Their semi‐fossorial nature further expands their foraging range, feeding on other invertebrates (e.g., Coleoptera), fruits and small vertebrates (Emmons and Feer 1997), potentially elevating their δ^15^N_c_ values. Despite marked differences in ecological, morphological and phylogenetic traits, along with contrasting habitat degradation sensitivity and population densities (McDonough and Loughry 2008), *D. novemcinctus* and *P. maximus* aligned at the intermediate level. This unexpected alignment suggests a shared ecological role between these highly distinct species, representing a second point of convergence.

At the top of the trophic structure, *E. sexcinctus* and *C. squamicaudis* exhibited highly ^15^N‐enriched diets, highlighting a second convergence point. The elevated δ^15^N_c_ value of *E. sexcinctus* aligns well with its omnivorous diet (McDonough and Loughry 2008) and carnivorous behaviour (e.g., Chatellenaz and Mestres 2023). In contrast, our findings provide new ecological insights into the poorly understood *Cabassous* genus, revealing that *C. squamicaudis* exhibits a more omnivorous diet and greater behavioural plasticity than previously recognised (McDonough and Loughry 2008). These results underscore the value of employing complementary approaches, such as stable isotope and dietary analyses, to deepen our understanding of species' ecological roles (Araújo et al. 2007). Such integrative frameworks are essential for uncovering ecological relationships and disentangling the trophic organisation of closely related species.

### 4.2 Spatial Dimension

#### 4.2.1 Stable Isotopes

Landscape composition, a proxy for resource availability, directly influenced xenarthran habitat use at individual and population levels, shaping their resource use and supporting our fourth hypothesis (H4). Consistent with previous findings, *P. maximus* and *D. novemcinctus* —forest‐dependent species (Magioli et al. 2023; Rodrigues and Chiarello 2018)—increased their consumption of C_3_ resources as forest cover rose, with this effect observed at the individual level for *P. maximus* and the population level for *D. novemcinctus* . Additionally, *D. novemcinctus* responded to increasing mixed cover, highlighting the importance of forest remnants within human‐modified landscapes as critical C_3_ food sources for population maintenance. The diet of *M. tridactyla* shifted toward C_3_ resources as savanna cover increased, strongly influencing its resource use at the individual level. Furthermore, both forest formations, which have higher mean δ^15^N than more open formations (Martinelli et al. 2021), positively influenced δ^15^N_c_ values, indicating a substantial contribution of forest‐derived prey at the individual level.

#### 4.2.2 Habitat Use

Habitat use aligned with and complemented the patterns observed through stable isotopes, as predicted by H4. The most consistent responses were observed for *P. maximus* and *M. tridactyla* , which increased their habitat use with rising forest cover at the individual level. For *E. sexcinctus* and *C. squamicaudis*—species adapted to open habitats—the positive response to increasing savanna cover may be attributed to their thermoregulation requirements, a pattern also expected for *M. tridactyla* (McNab 1985). This physiological need may further shape their resource use by increasing the consumption of prey feeding on arboreal resources (C_3_ plants), which could explain the individuals exhibiting mixed and strictly C_3_ diets, marking a third point of convergence. Additionally, *E. sexcinctus* responded negatively to increasing forest cover at the individual level, reinforcing its avoidance of dense forested habitats (McDonough and Loughry 2008).

In contrast, *D. novemcinctus* habitat use showed a neutral association with forests but displayed conflicting responses to anthropogenic covers: a negative association with mixed cover at the individual level, which contrasts with the positive response observed at the population level through stable isotopes, and opposing responses to pasture cover—positive at the population level and negative at the individual level. These inconsistencies reflect the trade‐offs inherent in human‐modified landscapes. While anthropogenic areas provide alternative food resources at the population level (Magioli et al. 2019; Manlick and Pauli 2020), foraging in these environments increases individual exposure to risks, including increased predation by native and domestic carnivores, road mortality, exposure to agrochemicals and poaching (Ferreguetti et al. 2016). Like *D. novemcinctus* , *P. maximus* also responded positively to pasture cover at the individual level. Although unexpected, this response aligns with the mixed and strictly C_4_ diets exhibited by some individuals of both species, reinforcing this third point of convergence. Furthermore, *E. sexcinctus* displayed a negative association with mixed cover at the individual level, mirroring the response of *D. novemcinctus* . This result underscores the complex dynamics of human‐modified landscapes, which can negatively affect even species considered common and resilient to habitat modification (McDonough and Loughry 2008). Overall, our findings emphasise the critical role of habitat availability in shaping species' diets and demonstrate how landscape composition may influence organisms' resource use across different spatial scales, highlighting the importance of integrating complementary approaches to capture subtle variations in species' ecological responses.

### 4.3 Temporal Dimension

Overlaps in activity patterns complemented responses from the trophic and spatial dimensions, adding another layer of similarity between the strictly nocturnal *P. maximus* and *D. novemcinctus* , and the primarily diurnal *E. sexcinctus* and *C. squamicaudis*, marking a fourth point of convergence within these species' pairs. Environmental temperatures strongly influence habitat use in organisms with low metabolic rates, adapted to open habitats and active during the day, such as the Xenarthra, with forest formations playing a pivotal role in thermoregulation (Lovegrove 2012). Consequently, activity patterns may influence habitat selection and resource use, as previously documented for *M. tridactyla* (Giroux et al. 2022) and corroborated by our study, which also supported similar responses for *C. squamicaudis* and *E. sexcinctus* . These findings underscore the intricate relationships between temporal patterns, habitat preferences and resource use (Van Scoyoc et al. 2024), highlighting their integrated role in shaping species' ecological niches.

### 4.4 Coexistence Patterns

Species coexistence may rely on niche partitioning (Chesson 2000), a pattern observed for most species' pairs in our study. However, two pairs exhibited convergence on all dimensions, suggesting they perform equivalent ecological roles and possibly compete for resources across space and time. As species similarity increases, the likelihood of stable coexistence decreases (Meszéna et al. 2006) since complete competitors cannot co‐occur (Hardin 1960). Consequently, additional mechanisms must explain their coexistence. For *D. novemcinctus* and *P. maximus* , morphological (e.g., body mass) and behavioural (e.g., home range) differences potentially minimise direct competition by shaping their responses to environmental conditions at different levels (population vs. individual, respectively). Additionally, *D. novemcinctus* is an important prey species for pumas ( *Puma concolor* ) and maned wolves ( *Chrysocyon brachyurus* ) (Magioli and Ferraz 2021; Rodrigues et al. 2007), which may regulate its population density, further reducing competitive pressure. In contrast, *C. squamicaudis* and *E. sexcinctus* share greater ecological similarities. A key distinction may lie in their foraging behaviour: *C. squamicaudis* is highly fossorial, likely feeding underground (Desbiez et al. 2018), whereas *E. sexcinctus* primarily forages above ground. This behavioural separation could reduce direct competition despite their similar dietary patterns. Therefore, we suggest incorporating other processes, such as predation, to evaluate additional layers of divergence/convergence, as observed in other taxa (Gurevitch et al. 2000).

## 5 Conclusions

The ecological niche is a fundamental concept for understanding species occurrence, coexistence and spatial and temporal segregation (Chase and Leibold 2003). However, many studies assessing niche divergence employ analytical approaches that reduce comparisons to a single dimension, which may limit ecological insights (Ascanio et al. 2024). Our integrative framework underscores the importance of complementary approaches in investigating niche convergences and divergences, testing ecological theories and uncovering hidden relationships within guild dynamics and ecosystem interactions. Furthermore, our findings highlight the urgent need to expand natural history research, as detailed ecological data are essential for robust assessments and provide a critical foundation for conservation efforts to mitigate population declines.

## Author Contributions

M.M., A.L.J.D., and A.G.C. designed the research. V.A., N.A., and A.L.J.D. collected data. M.M., M.Z.M., E.A.R.C. Jr, and A.G.C. analysed data. M.M. performed research and drafted the manuscript. All authors contributed substantially to the manuscript development.

## Conflicts of Interest

The authors declare no conflicts of interest.

## Peer Review

The peer review history for this article is available at https://www.webofscience.com/api/gateway/wos/peer‐review/10.1111/ele.70173.

## Supporting information


Data S1.

## Data Availability

Data and codes are available at Zenodo (https://doi.org/10.5281/zenodo.14630208).
