# Supplementary material for: Divergence Across Niche Dimensions Reveals Species' Ecological Roles
Source: Ecol Lett. 2025 Jul 11;28(7):e70173. doi: 10.1111/ele.70173 (PMC12246884; doi:10.1111/ele.70173)
Supplement: Supplementary file 1 — Data S1. [file ELE-28-0-s001.pdf]

Supplementary Information for:

Divergence across niche dimensions reveals species' ecological roles

Marcelo Magioli, Vinícius Alberici, Elildo A.R. Carvalho Jr, Nina Attias, Katia Maria Paschoaletto Micchi de Barros Ferraz, Marcelo Zacharias Moreira, Arnaud L. J. Desbiez, Adriano Garcia Chiarello

Summary

S1: Data collection 2
Biological models 2
Data collection 2
Hair sample collection 2
Camera trapping 3
Interpreting isotopic values 3
Figure S1 4
Figure S2 5
Figure S3 6
Figure S4 7
S2 Landscape metrics: 8
Landscape variables 8
Species home range 8
Table S1 8
S3 Data analysis: 9
Trophic dimension 9
Trophic discriminant factors 9
Resource use 9
Feeding habits 9
Spatial dimension 10
Resource use 10
Habitat use 10
Table S2 10
Table S3 11
Table S4 13
Table S5 14
Table S6 14
S4 Supporting results: 15
Assumptions check for ANOVA tests 15
Table S7 15
Table S8 15
Table S9 16
Table S10 16
Table S11 16
Table S12 17
Assumptions check for linear regression models 17
Figure S5 17
Figure S6 18
Figure S7 18
Figure S8 19
Figure S9 19
Figure S10 20
Figure S11 20
Supporting references 21

S1: Data collection

Biological models

The insectivorous xenarthrans, our focal group in this study, belong to two orders: Pilosa (Myrmecophagidae) and Cingulata (Dasypodidae and Chlamyphoridae). Within Pilosa, the family Myrmecophagidae includes two species. The giant anteater (*Myrmecophaga tridactyla*) (Supplementary Figure S2A) is a large (~30 kg) terrestrial species, and strictly myrmecophagous, primarily consuming ants and termites. It inhabits both open vegetation formations, such as the Cerrado, and dense forests, including the Atlantic Forest and Amazon (Gaudin *et al.* 2018). The species is classified as vulnerable to extinction in Brazil (MMA 2022) and globally (IUCN 2024). The southern tamandua (*Tamandua tetradactyla*) (Supplementary Figure S2B), a medium-sized (~5.2 kg) scansorial mammal, is widespread across South American forests and arboreal savannas, maintaining a strictly myrmecophagous diet (Hayssen 2011). These myrmecophagids share key morphological traits, exhibit similar dietary composition (Gaudin *et al.* 2018; Hayssen 2011), and are closely related phylogenetically (Gibb *et al.* 2016).

From the Cingulata order, the nine-banded armadillo (*Dasypus novemcinctus*) (Supplementary Figure S2C) is the sole representative of the Dasypodidae family included in this study. This semi-fossorial, medium-sized mammal (~3.2 kg) has an insectivorous/omnivorous diet and is found in forest formations, ranging from pristine to degraded habitats (McBee & Baker 1982). Remarkably, it appears to adapt well to human-modified landscapes (Rodrigues & Chiarello 2018). A recent taxonomic revision has divided the species into four distinct species across its previously extensive distribution (Barthe *et al.* 2024).

The remaining Cingulata species belong to the Chlamyphoridae family, which includes: six-banded armadillo (*Euphractus sexcinctus*) (Supplementary Figure S2D), a semi-fossorial, insectivorous/omnivorous, medium-sized species (~5.4 kg), adapted to open vegetation formations (Redford & Wetzel 1985), and noted for its peculiar carnivorous behavior (Chatellenaz & Mestres 2023); the southern naked-tailed armadillo (*Cabassous squamicaudis*) (Supplementary Figure Fig. S2E), a subterranean (Desbiez *et al.* 2018) medium-sized mammal (~2.0 kg), recently revalidated as a species, primarily inhabits savanna formations (Feijó & Anacleto 2021) and exhibits an insectivorous/omnivorous diet (Bonato 2002), though significant knowledge gaps remain about its biology and ecology (Loughry *et al.* 2015); and the giant armadillo (*Priodontes maximus*) (Supplementary Figure Fig. S2F), the largest extant armadillo species (~26.8 kg), displaying semi-fossorial behavior (Carter *et al.* 2016), an insectivorous/omnivorous diet (Nascimento *et al.* 2024), a preference for forested habitats (Desbiez *et al.* 2020; Magioli *et al.* 2023), and classified as vulnerable to extinction in Brazil (MMA 2022) and globally (IUCN 2024). Although all three species are chlamyphorids, *C. squamicaudis* and *P. maximus* are more closely related phylogenetically (Gibb *et al.* 2016), sharing morphological and behavioral similarities (Wetzel 1985b, a), and are thought to have similar dietary preferences (Anacleto 2007).

Data collection

In this study, we utilized two primary datasets: the first consists of hair samples from insectivorous xenarthrans collected in the Cerrado (*C. squamicaudis*, *D. novemcinctus*, *E. sexcinctus*, *M. tridactyla*, and *T. tetradactyla*) and Pantanal landscapes (*P. maximus*) for isotopic analysis; the second comprises camera trap data from the Cerrado landscape (for all six species), used to perform occupancy models and analyze temporal activity patterns.

Hair sample collection

The samples used for isotopic analysis from the species in the Cerrado biome were all collected during the same period in 2019 to minimize seasonal dietary variation. Despite *P. maximus* samples being collected in the Pantanal, mean carbon isotopic values of the vegetation in both biomes are similar [Cerrado: -28.9‰; Pantanal: -30.1‰; (Martinelli *et*

al. 2021)]. Additionally, the landscape composition regarding the proportions of C_3 and C_4 plants is comparable between the two areas (Cerrado: 69% of C_4 and 31% of C_3 plants; Pantanal: 76% of C_4 and 24% of C_3 plants) (Supplementary Figure S1), which allows for meaningful comparison among species between landscapes.

Camera trapping

Each site was equipped with three Reconyx® HyperFire™ camera traps close to the center of the site: one placed inside the native vegetation remnant, another along a trail (e.g., farm or unpaved road), and the third facing a *P. maximus* burrow. In the absence of trails or burrows, the remaining cameras were positioned inside the remnants. An average effort of 87.74 trap-days per site was obtained. As the three cameras per site were not spatially independent, we grouped them as a single camera, considering as independent records those of the same species taken at the same site with a minimum interval of one hour. The landscape composition of the 10-km buffer zones is similar between the two road sections, enabling comparison.

Interpreting isotopic values

Stable carbon isotopes ($\delta^{13}C$) in terrestrial environments are associated with land cover [e.g., forest formations, open formations (e.g., grasslands), or anthropogenic areas (e.g., agriculture, pasture)], as plants vary in their isotopic values according to the photosynthetic cycle (e.g., C_3 and C_4). Forests are dominated by C_3 plants (e.g., trees, shrubs), while C_4 plants are more commonly associated with open formations (e.g., grasslands), pasture, and C_4 crops (e.g., sugarcane, maize). Therefore, isotopic values markedly differ between C_3 and C_4 plants, with neotropical C_3 plants ranging from -38 to -24‰, while C_4 plants range from -15 to -11‰ (Martinelli *et al.* 2021). In this sense, with $\delta^{13}C$ values, it is possible to infer the origin of the food sources consumed by organisms (e.g., forest vs agriculture), their foraging preferences, and habitat use. Stable nitrogen isotopes ($\delta^{15}N$) are employed to uncover dietary patterns and trophic processes, and tend to be enriched by ~3‰ at each level in the trophic chain (Post 2002), increasing from primary consumers (smaller values) to top predators (higher values). Therefore, nitrogen isotopes are a reliable tool for understanding variation in diet composition among species, trophic guilds, or functional groups, and confer insights on trophic structuring (Crawford *et al.* 2008).

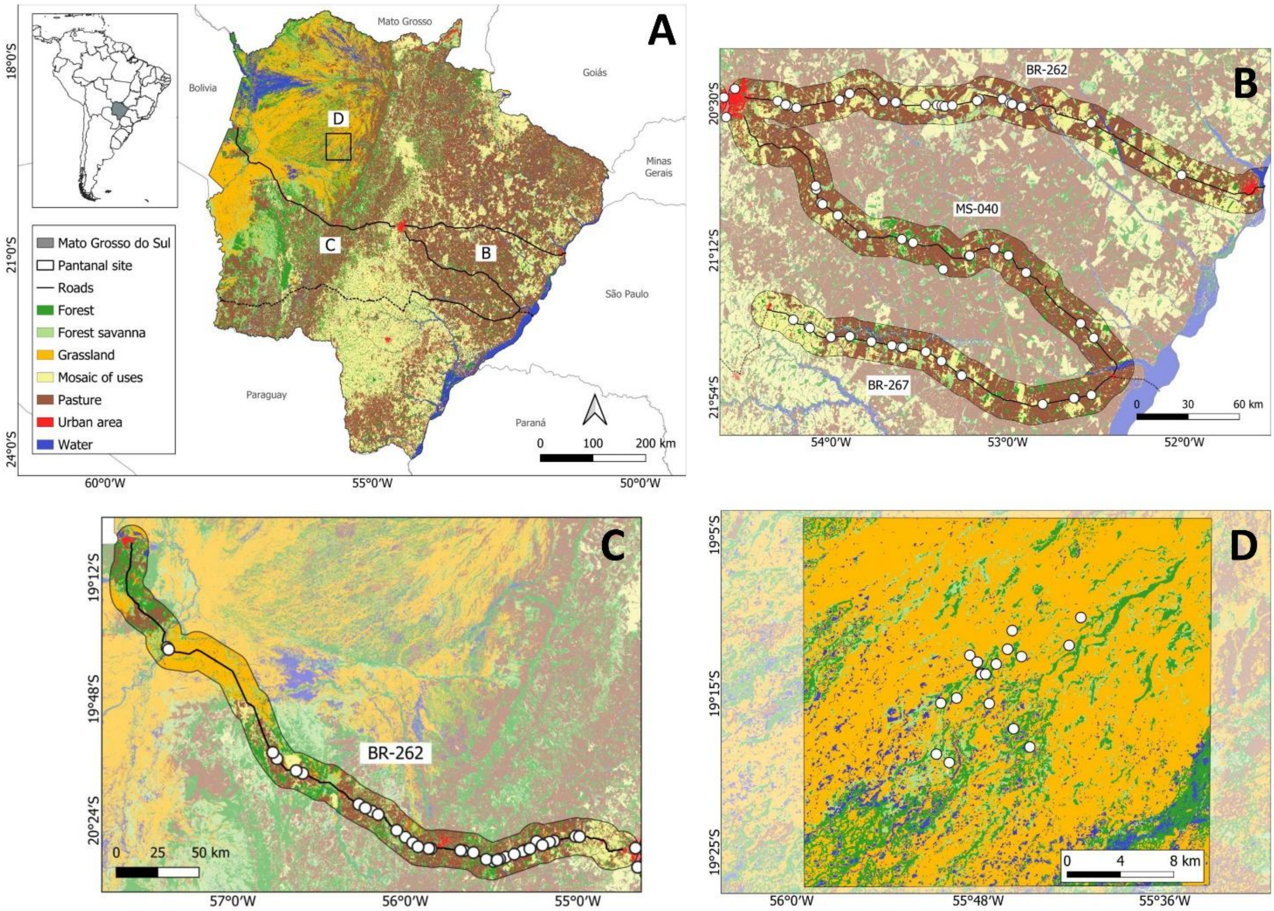

Figure S1

Location of the study areas and where hair samples (white dots) from anteaters and armadillos were collected in the Cerrado and Pantanal landscapes in Mato Grosso do Sul, Brazil. A) Mato Grosso do Sul state. B) Zoomed-in view of the landscapes along BR-262 (east portion), BR-267, and MS-040 highways. C) Zoomed-in view of the landscape along the west portion of the BR-262 highway. D) Pantanal site in the Nhecolândia subregion. The main land uses are depicted according to Projeto MapBiomas (Projeto MapBiomas 2023).

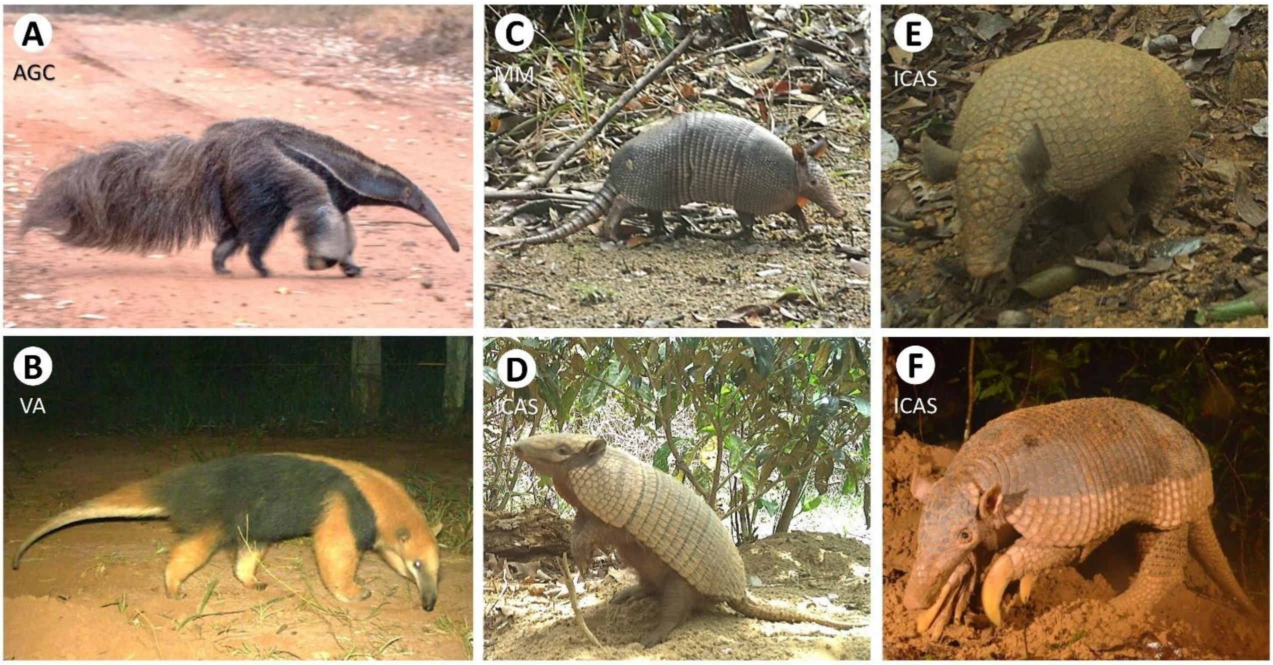

Figure S2

Photographs of the studied anteater and armadillo species. A) *Myrmecophaga tridactyla*; B) *Tamandua tetradactyla*; C) *Dasypus novemcinctus*; D) *Euphractus sexcinctus*; E) *Cabassous squamicaudis*; F) *Priodontes maximus*. Author and institutional credits are displayed in the photographs.

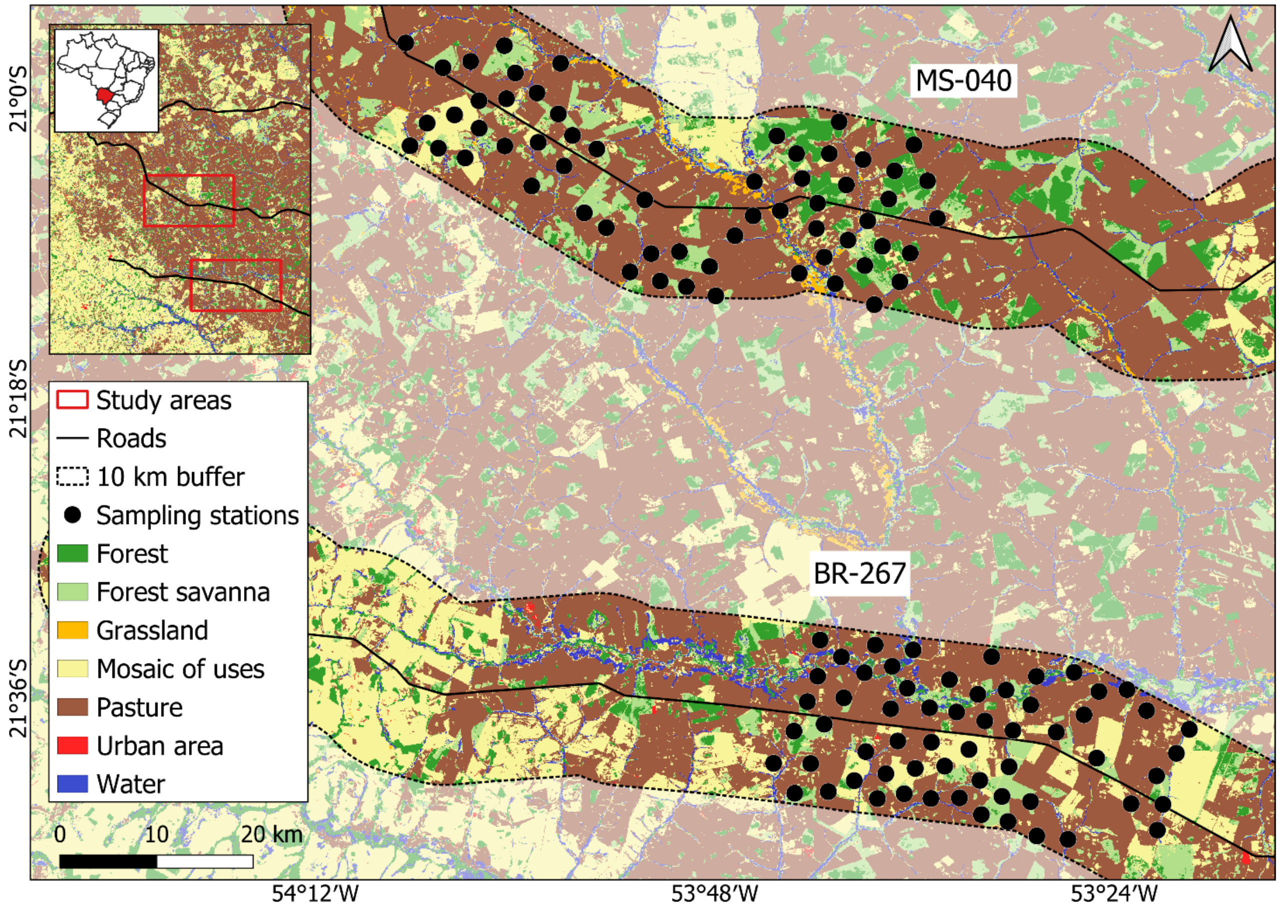

Figure S3

Location of the camera trap stations in MS-040 and BR-267 highways in Mato Grosso do Sul, Brazil, depicting the main land uses (Projeto MapBiomas 2023).

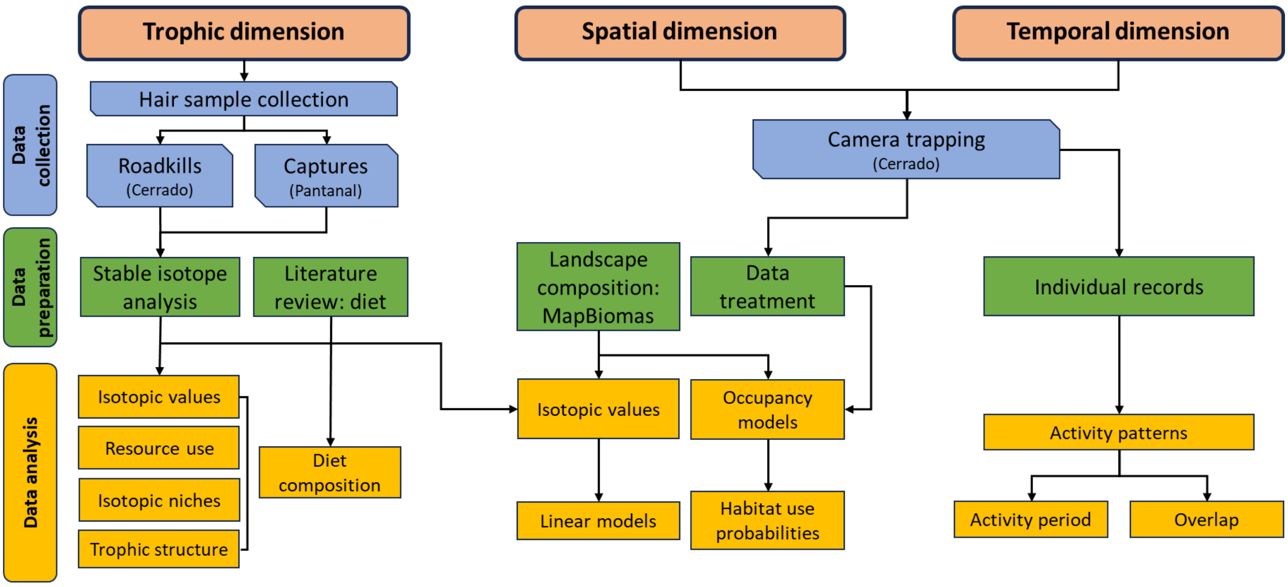

Figure S4

Study workflow depicting data collection, preparation, and analyses employed.

S2 Landscape metrics:

Landscape variables

From the available categories in the MapBiomas land cover map (Projeto MapBiomas 2023), we used histograms to select those with sufficient variation across the sites, including forest formations [semideciduous forests (legend code 3) and savanna (legend code 4)], grasslands (legend code 12), pastures (legend code 15), and mosaic of uses (legend code 21) (https://brasil.mapbiomas.org/wp-content/uploads/sites/4/2023/08/Legenda-Colecao-8-LEGEND-CODE.pdf). We note that grasslands were only considered for the Pantanal landscape because their variation in the Cerrado landscape is negligible.

Species home range

To account for the distinct home ranges of the studied species, we compiled home range data from the literature to calculate landscape variables at ecologically meaningful scales for each species. In December 2023, we performed a literature search in Web of Science, Scopus, and Google Scholar for studies presenting home range information of the target species. We searched for indexed and non-indexed peer-reviewed articles and grey literature, resulting in the compilation of studies with home range information for all six species (Supplementary Table S1).

Table S1

Estimated and adopted home ranges (in hectares) and buffer size (in meters) for the anteater and armadillo species.

**Species**	**Estimated (ha)**	**Adopted (ha)**	**Buffer size (m)**	**References**
Cabassous squamicaudis	380	380	1100	Desbiez *et al.* 2018
*Dasypus novemcinctus**	2 to 20	20 (max)	252.4	Loughry & McDonough 2013
Euphractus sexcinctus	17 ± 23	40 (max)	357	Medri 2008
Myrmecophaga tridactyla	680	680	1471	Noonan *et al.* 2022
Priodontes maximus	2500	2500	2821	Desbiez *et al.* 2020
Tamandua tetradactyla	15 to 489	252 (avg)	895	Araújo 2013

* *Dasypus novemcinctus* was recently divided into four species (Barthe *et al.* 2024), limiting the amount of available information for the species. Most information on the biology and ecology of the genus *Dasypus* is from studies in the United States, which is now represented by *Dasypus mexicanus*. In the face of the limited amount of information for *D. novemcinctus* in South America concerning their home range, we utilized available information from *D. mexicanus* to determine the buffer size for the calculations of landscape composition.

S3 Data analysis:

Trophic dimension

Trophic discriminant factors

To estimate the trophic discriminant factors for each species, we ran three chains with 1,200,000 Markov Chain Monte Carlo (MCMC) iterations, including a burn-in period of 200,000 and a thinning rate of 500. Parameter convergence was assessed using the Gelman-Rubin diagnostic (Gelman & Shirley 2011) and visual inspection of trace plots. All chains exhibited R-hat values < 1.1 for all parameters, indicating convergence.

Resource use

To determine the origin of food items consumed by insectivorous Xenarthra – mainly invertebrates feeding on C_3 and C_4 plants – we employed a simple mixed model that interpolates hair samples' $\delta^{13}C_c$ values with the mean reference values of the different vegetation types (C_3 and C_4 plant photosynthetic cycles), allowing the calculation of the proportion of C_3/C_4 carbon in each sample (see Magioli *et al.* 2014). Reference isotopic values of the vegetation for the models were specific to the Cerrado and Pantanal biomes (Martinelli *et al.* 2021). Therefore, we calculated the carbon source in each sample (%C_3) using the following equation:

$$C_3\ carbon\ incorporated\ (\%)\ = \frac{\delta^{13}C_c\ sample - \delta^{13}C_{mean}\,C_4\ vegetation}{\delta^{13}C_{mean}\ C_3\ vegetation\ -\ \delta^{13}C_{mean}\,C_4\ vegetation} * 100$$

We used the carbon isotopic values of -28.9‰ and -30.1‰ to represent C_3 plants (e.g., forest remnants) in the Cerrado and Pantanal, respectively, as end-members in our model, based on data from Martinelli *et al.* (2021). For C_4 plants (e.g., agricultural areas, grasslands), we used a carbon isotopic value of -12.0‰ V-PDB for both biomes, based on values obtained for pasture and sugarcane in previous studies (Magioli *et al.* 2014, 2019). After calculating the proportion of C_3/C_4 carbon, we classified samples into three groups: (1) C_3-based – individuals that preferentially consumed C_3 items (> 70% of C_3 carbon; Cerrado, $\delta^{13}C_c$ = -28.9 to -23.8‰; Pantanal, $\delta^{13}C_c$ = -30.1 to -24.7‰); (2) Mixed – individuals that used both C_3 and C_4 food items (from 30 to 70% of C_3 carbon; Cerrado, $\delta^{13}C_c$ = -23.7 to -17.1‰; Pantanal, $\delta^{13}C_c$ = -24.6 to -17.5‰); C_4-based – individuals that mainly consumed C_4 items (< 30% of C_3 carbon; Cerrado, $\delta^{13}C_c$ = -17.0 to -12.0‰; Pantanal, $\delta^{13}C_c$ = -17.4 to -12.0‰).

Feeding habits

To conduct the literature search on the species' feeding habits, we used various combinations of keywords in English, Portuguese, and Spanish, including both scientific and common names of species, along with terms such as "diet" and "feeding habit". We searched for indexed and non-indexed peer-reviewed articles and grey literature, and explored references cited within selected studies for further relevant sources. For the quantification of the diet, although some studies presented more than five samples, they did not have enough information for the calculations (N = 9), and were excluded, totaling 20 studies for analysis. Even though there are more diet studies available for *Dasypus novemcinctus*, the species was recently divided into four species (Barthe *et al.* 2024), preventing the comparison of its diet throughout the previous wide distribution, particularly studies in the United States, which now belong to *Dasypus mexicanus*.

Spatial dimension

Resource use

For the variable selection procedure, we ran three chains with 100,000 Markov Chain Monte Carlo (MCMC) iterations, including a burn-in period of 50,000 and a thinning rate of 100. Parameter convergence was assessed using the Gelman-Rubin diagnostic (Gelman & Shirley 2011) and visual inspection of trace plots. All chains exhibited R-hat values < 1.1 for all parameters, indicating convergence.

Habitat use

The Bayesian single-species occupancy models use detection/non-detection data to estimate occupancy probability while accounting for imperfect detection (Kéry & Schaub 2012; MacKenzie *et al.* 2017; Royle & Dorazio 2008). We assumed the probability of occupancy as habitat use. In these models, the latent state variable "occurrence of a given species at site i" is specified as a Bernoulli outcome governed by the habitat use probability (ψ) of the species at site i: $z_i \sim \text{Bern}(\psi_i)$. To account for imperfect detection (r), the observation process is modeled as a binomial outcome governed by z_i multiplied by the detection probability of the species at site i during sampling occasion k: $y_i \sim B(z_i \times r_{i,k})$ (Kéry & Schaub 2012). We considered five consecutive days as one sampling occasion. The covariates for r and ψ were implemented using logit link functions (Kéry & Schaub 2012). Non-informative priors were applied to all parameters. For the variable selection procedure, we ran three chains with 100,000 Markov Chain Monte Carlo (MCMC) iterations, including a burn-in period of 50,000 and a thinning rate of 100. For the final models, we ran three chains with MCMC iterations varying between 100,000 and 300,000, including a burn-in period varying between 50,000 and 150,000, and a thinning rate varying between 100 and 300. The variation in the parameters adopted for the final models among species was necessary to achieve convergence. Parameter convergence was assessed using the Gelman-Rubin diagnostic (Gelman & Shirley 2011) and visual inspection of trace plots. All chains exhibited R-hat values < 1.1 for all parameters, indicating convergence.

Table S2

Estimated mean discrimination factors and standard deviation (SD) for stable carbon ($\Delta^{13}C$) and nitrogen ($\Delta^{15}N$) isotopes from hair of anteater and armadillo species.

Species	$\Delta^{13}C$ (‰)	SD	$\Delta^{15}N$ (‰)	SD
Cabassous squamicaudis	2.1	1.9	3.3	1.6
Dasypus novemcinctus	2.1	2.0	3.3	1.6
Euphractus sexcinctus	2.8	1.9	3.6	1.6
Myrmecophaga tridactyla	2.1	2.0	3.3	1.6
Priodontes maximus	2.1	2.0	3.2	1.6
Tamandua tetradactyla	2.1	2.0	3.3	1.6

Table S3

Methods of assessments, number of samples, biome, and localities of the studies on the feeding habits of anteater and armadillo species throughout their distribution. Studies are organized chronologically.

Species	Study	Methods	Samples	Biome	Localities
Cabassous squamicaudis	(Bonato 2002)	Feces, stomach content	28	Cerrado	São Paulo, Brazil
	(Anacleto 2007)	Feces	1	Cerrado	Mato Grosso, Brazil
*Dasypus novemcinctus**	(Anthony-Mathews 1977)	Stomach content	2	Cerrado	Mato Grosso, Brazil
	(Redford 1986)	Stomach content	10	Cerrado	Goiás, Brazil
	(Vaz *et al.* 2012)	Feces, stomach content	14	Caatinga	Piauí, Brazil
	(Pasa *et al.* 2020)	Stomach content	3	Atlantic Forest	Rio Grande do Sul, Brazil
	(Yone *et al.* 2020)	Stomach content	18	Atlantic Forest	Rio de Janeiro, Brazil
Euphractus sexcinctus	(Schaller 1983)	Stomach content	10	Pantanal	Mato Grosso, Brazil
	(Redford 1985)	Stomach content	-	Chaco	Paraguay
	(Bonato 2002)	Feces	22	Cerrado	Mato Grosso, Brazil
	(Dalponte & Tavares-Filho 2004)	Stomach content	12	Cerrado	São Paulo and Mato Grosso, Brazil
	(Anacleto 2007)	Feces	8	Pantanal	Mato Grosso do Sul, Brazil
	(Medri 2008)	Feces	28	Cerrado	Mato Grosso do Sul, Brazil
	(Vaz *et al.* 2012)	Feces, stomach content	3	Caatinga	Piauí, Brazil
	(Zambrini 2015)	Feces	8	Pantanal	Mato Grosso do Sul, Brazil
	(Pasa *et al.* 2020)	Stomach content	3	Atlantic Forest	Rio Grande do Sul, Brazil
Myrmecophaga tridactyla	(Montgomery 1985)	Stomach content	28	Llanos	Venezuela
	(Shaw *et al.* 1985)	Observation	58	Llanos	Venezuela
	(Redford 1986)	Feces, stomach content, observation	-	Several	Brazil, Paraguay, Venezuela
	(Drumond 1992)	Observation	316	Cerrado	Minas Gerais, Brazil
	(Medri *et al.* 2003)	Observation	50	Pantanal	Mato Grosso do Sul, Brazil
	(Vaz *et al.* 2012)	Feces	1	Caatinga	Piauí, Brazil
	(Sandoval-Gómez *et al.* 2012)	Feces, stomach content	12	Amazon	Colombia
	(Braga *et al.* 2014)	Feces	24	Atlantic Forest	Paraná, Brazil
	(Gallo *et al.* 2017)	Feces	NA	Chaco	Argentina
	(Fuster *et al.* 2018)	Feces, stomach content	5	Chaco	Argentina
	(Jiménez *et al.* 2018)	Feces	22	Chaco	Corrientes, Argentina

Species	Study	Methods	Samples	Biome	Localities
	(Silva *et al.* 2023)	Feces, stomach content	31	Cerrado	Mato Grosso do Sul, Brazil
Priodontes maximus	(Anacleto & Marinho-Filho 2001)	Feces	25	Cerrado	Minas Gerais, Brazil
	(Pitman *et al.* 2004)	Observation	15	Amazon	Peru
	(Anacleto 2007)	Feces	8	Cerrado	Mato Grosso, Brazil
	(Nascimento *et al.* 2024)	Feces	113	Pantanal	Mato Grosso do Sul, Brazil
Tamandua tetradactyla	(Montgomery 1985)	Feces, stomach content	19	Llanos	Venezuela
	(Sousa & Messias 2006)	Stomach content	4	Amazon	Paraná, Brazil
	(Ferreira *et al.* 2007)	Stomach content	11	Mata Atlântica	Roraima, Brazil
	(Vaz *et al.* 2012)	Feces, stomach content	4	Caatinga	Piauí, Brazil
	(Gallo *et al.* 2017)	Stomach content	4	Mata Atlântica, Chaco	Argentina
	(Silva 2019)	Stomach content	8	Mata Atlântica	São Paulo, Rio de Janeiro
	(Pasa *et al.* 2020)	Stomach content	2	Atlantic Forest	Rio Grande do Sul, Brazil
	(Deloss *et al.* 2024)	Stomach content	16	Pampas	Rio Grande do Sul, Brazil

* Although there are more diet studies available for *Dasypus novemcinctus*, the species was recently divided into four species (Barthe *et al.* 2024), preventing the comparison of its diet throughout the previous wide distribution, particularly of studies in the United States, which is now represented by *Dasypus mexicanus*.

Table S4

Frequency and percentage of occurrence of food items in the diet of anteaters and armadillos throughout their distribution, depicting the contribution of invertebrates (highlighting ants, termites, and coleopterans), vertebrates, and plant material. Studies are organized chronologically.

Species	Study	Diet	Frequency of occurrence (%)						Percentage of occurrence (%)					
			Invert.	Ants	Termites	Coleoptera	Vert.	Plants	Invert.	Ants	Termites	Coleoptera	Vert.	Plants
Cabassous squamicaudis	(Bonato 2002)	In/Om	100.0	96.4	96.4	46.4	-	46.4						
Dasypus novemcinctus	(Vaz *et al.* 2012)	In/Om	100.0	71.4	92.9	78.6	7.1	71.4						
	(Yone *et al.* 2020)			94.0	0.0	77.0	11.0	89.0						
Euphractus sexcinctus	(Bonato 2002)	In/Om	100.0	90.1	59.1	72.7	22.7	95.4						
	(Dalponte & Tavares-Filho 2004)								62.6	22.0	1.0	30.7	3.9	32.6
	(Anacleto 2007)								98.4	73.2	20.3	1.7	1.1	-
	(Medri 2008)		100.0	71.4	21.4	89.3	10.7	35.7						
Myrmecophaga tridactyla	(Shaw *et al.* 1985)	Myr	100.0	88.0	12.0	-	-	-						
	(Montgomery 1985)		100.0	96.0	4.0	-	-	-						
	(Redford 1986)		100.0	11.0	89.0	-	-	-						
	(Drumond 1992)		100.0	45.0	55.0	-	-	-						
	(Medri *et al.* 2003)		100.0	81.0	19.0	-	-	-						
	(Braga *et al.* 2014)		100.0	73.7	26.3	-	-	-						
	(Jiménez *et al.* 2018)		100.0	99.5	0.5	-	-	-						
	(Silva *et al.* 2023)		100.0	29.2	69.9	-	-	0.9						
Priodontes maximus	(Anacleto & Marinho-Filho 2001)	In/Om	100.0	36.0	96.0	-	-	28.0						
	(Pitman *et al.* 2004)		100.0	1.0	92.0			20.0						
	(Anacleto 2007)								100.0	27.5	61.4	11.1	-	-
	(Nascimento *et al.* 2024)		100.0	72.4	100.0	-	6.8	41.4						
Tamandua tetradactyla	(Montgomery 1985)	Myr	100.0	49.0	51.0	-	-	-						
	(Ferreira *et al.* 2007)		100.0	77.5	22.5	-	-	-						
	(Silva 2019)		100.0	100.0	87.5	25	-	-						
	(Deloss *et al.* 2024)		100.0	100.0	62.5	12.5	-	-						

Table S5

Linear regression model (lm) formulas relating isotopic values ($\delta^{13}C_c$ or $\delta^{15}N_c$) to landscape composition variables selected by the stepwise procedure for anteaters and armadillos in the Cerrado and Pantanal landscapes, Mato Grosso do Sul, Brazil,. Species marked with a hyphen indicate that the null model was selected. Categories include forest (for), savanna (sav), and mosaic of uses (mix). The numbers following the underscore represent the scale of effect. Model assumptions were verified with the performance package (Lüdecke *et al.* 2021).

**Species**	**$\delta^{13}C_c$ (‰)**	**$\delta^{15}N_c$ (‰)**
Cabassous squamicaudis	lm ($\delta^{13}C_c \sim$ for_500)	lm ($\delta^{15}N_c \sim$ for_500)
Dasypus novemcinctus	lm ($\delta^{13}C_c \sim$ mix_2000)<br>lm ($\delta^{13}C_c \sim$ for_2000)	-
Euphractus sexcinctus	-	-
Myrmecophaga tridactyla	lm ($\delta^{13}C_c \sim$ sav_2000)	lm ($\delta^{15}N_c \sim$ for_1000^2)<br>lm ($\delta^{15}N_c \sim$ sav_2000)
Priodontes maximus	lm ($\delta^{13}C_c \sim$ for_2500)	-
Tamandua tetradactyla	-	-

Table S6

Occupancy model formulas used to assess the effect of landscape composition on the occupancy (ψ) and detection (r) of anteaters and armadillos in the Cerrado and Pantanal landscapes, Mato Grosso do Sul, Brazil. The variables presented were selected by the stepwise procedure. Categories include forest (for), savanna (sav), and mosaic of uses (mix). The numbers following the underscore represent the scale of effect; *i* represents each camera trap sampling site.

**Species**	**Models**
Cabassous squamicaudis	$\text{logit}(\psi_i) = \alpha0_i + \alpha1_i\ \text{sav\_500}_i$<br>$\text{logit}(r_i) = \beta0_i + \beta1_i\ \text{sav\_500}_i$
Dasypus novemcinctus	$\text{logit}(\psi_i) = \alpha0_i + \alpha1_i\ \text{mix\_20}_i + \alpha2_i\ \text{pas\_2000}_i + \alpha3_i\ \text{pas\_20}_i$<br>$\text{logit}(r_i) = \beta0_i + \beta1_i\ \text{mix\_20}_i + \beta2_i\ \text{pas\_2000}_i + \beta3_i\ \text{pas\_20}_i$
Euphractus sexcinctus	$\text{logit}(\psi_i) = \alpha0_i + \alpha1_i\ \text{sav\_500}_i + \alpha2_i\ \text{mix\_2000}_i + \alpha3_i\ \text{for\_500}_i$<br>$\text{logit}(r_i) = \beta0_i + \beta1_i\ \text{sav\_500}_i + \beta2_i\ \text{mix\_2000}_i + \beta3_i\ \text{for\_500}_i$
Myrmecophaga tridactyla	$\text{logit}(\psi_i) = \alpha0_i + \alpha1_i\ \text{for\_500}_i + \alpha2_i\ \text{mix\_500}_i$<br>$\text{logit}(r_i) = \beta0_i + \beta1_i\ \text{for\_500}_i + \beta2_i\ \text{mix\_500}_i$
Priodontes maximus	$\text{logit}(\psi_i) = \alpha0_i + + \alpha1_i\ \text{for\_500}_i + \alpha2_i\ \text{pas\_500}_i$<br>$\text{logit}(r_i) = \beta0_i + \beta1_i\ \text{for\_500}_i + \beta2_i\ \text{pas\_500}_i$
Tamandua tetradactyla	$\text{logit}(\psi_i) = \alpha0_i + \alpha1_i\ \text{for\_500}_i$<br>$\text{logit}(r_i) = \beta0_i + \beta1_i\ \text{for\_500}_i$

S4 Supporting results:

Assumptions check for ANOVA tests

For the ANOVA test among isotopic values, the assumptions of homogeneity (Levene test; $\delta^{13}C_c$: $F = 0.95$, $p = 0.45$; $\delta^{15}N_c$: $F = 0.76$, $p = 0.58$) and residuals normality (Shapiro-Wilk test; $\delta^{13}C_c$: $W = 0.98$, $p = 0.10$; $\delta^{15}N_c$: $W = 0.99$, $p = 0.28$) were met for both isotopes.

Table S7

Minimum, maximum, average, and range of isotopic values ($\delta^{13}C_c$ and $\delta^{15}N_c$) for anteaters and armadillos in the Cerrado and Pantanal landscapes, Mato Grosso do Sul, Brazil.

Species	$\delta^{13}C_c$ (‰)				$\delta^{15}N_c$ (‰)			
	Min	Max	Mean	Range	Min	Max	Mean	Range
Cabassous unicinctus	-24.0	-10.7	-15.7	13.3	4.2	8.5	6.3	4.3
Dasypus novemcinctus	-24.9	-14.3	-18.9	10.6	2.2	6.1	4.3	3.9
Euphractus sexcinctus	-24.4	-12.6	-16.8	11.8	4.4	7.9	5.9	3.5
Myrmecophaga tridactyla	-20.0	-10.3	-13.8	9.7	1.0	5.3	2.9	4.3
Priodontes maximus	-23.5	-14.9	-19.6	8.6	2.5	8.6	4.4	6.1
Tamandua tetradactyla	-25.5	-12.7	-19.8	12.8	-0.4	5.6	2.9	6.0

Table S8

Comparison between pairs of anteaters and armadillos in the Cerrado and Pantanal landscapes, Mato Grosso do Sul, Brazil, resulting from the perMANOVA analysis. Significant differences are highlighted in red. MT = *Myrmecophaga tridactyla*; TT = *Tamandua tetradactyla*; DN = *Dasypus novemcinctus*; CS = *Cabassous squamicaudis*; ES = *Euphractus sexcinctus*; PM = *Priodontes maximus*.

Pairs	Sum-Squared	F-value	R^2	Adj-p
MT vs TT	354.05	32.57	0.46	0.02
MT vs DN	281.56	32.35	0.46	0.02
MT vs CS	156.58	12.73	0.25	0.02
MT vs ES	182.41	17.70	0.32	0.02
MT vs PM	349.15	45.55	0.55	0.02
TT vs DN	27.25	2.35	0.06	1.00
TT vs CS	285.21	18.76	0.33	0.02
TT vs ES	175.83	13.31	0.26	0.02
TT vs PM	20.06	1.88	0.05	1.00
DN vs CS	145.66	11.17	0.23	0.05
DN vs ES	68.54	6.21	0.14	0.14
DN vs PM	4.79	0.57	0.02	1.00
CS vs ES	15.07	1.03	0.03	1.00
CS vs PM	190.43	15.71	0.30	0.02
ES vs PM	99.82	9.91	0.21	0.05

Table S9

Overlap of the isotopic niche at 95% contour of anteaters and armadillos in the Cerrado and Pantanal landscapes, Mato Grosso do Sul, Brazil. Overlap values above 50% are highlighted in red.

Species	*M. tridactyla*	*T. tetradactyla*	*D. novemcinctus*	*C. squamicaudis*	*E. sexcinctus*	*P. maximus*
M. tridactyla	-	58.4	53.7	44.5	38.2	45.0
T. tetradactyla	32.8	-	46.8	43.9	36.8	36.1
D. novemcinctus	48.1	74.7	-	75.4	65.3	68.5
C. squamicaudis	19.7	34.6	37.3	-	72.6	33.4
E. sexcinctus	23.0	39.6	44.0	98.9	-	35.8
P. maximus	47.2	67.4	80.1	79.0	62.2	-
**Niche size (‰²)**	44.6	79.4	49.8	100.8	73.9	42.5

Table S10

Overlap of the isotopic niche at 50% contour of anteaters and armadillos in the Cerrado and Pantanal landscapes, Mato Grosso do Sul, Brazil. Overlap values above 50% are highlighted in red.

Species	*M. tridactyla*	*T. tetradactyla*	*D. novemcinctus*	*C. squamicaudis*	*E. sexcinctus*	*P. maximus*
M. tridactyla	-	1.4	0.7	0.0	0.0	0.0
T. tetradactyla	0.8	-	9.0	0.0	0.0	25.8
D. novemcinctus	0.6	14.7	-	3.2	29.2	46.6
C. squamicaudis	0.0	0.0	1.3	-	49.1	0.0
E. sexcinctus	0.0	0.0	15.9	65.6	-	3.1
P. maximus	0.0	45.5	50.5	0.0	6.2	-
**Niche size (‰²)**	10.3	18.6	11.4	28.2	21.1	10.6

Table S11

Non-significant relationships between isotopic values ($\delta^{13}C_c$ or $\delta^{15}N_c$) and landscape composition variables at different scales of effect for anteaters and armadillos in the Cerrado and Pantanal landscapes, Mato Grosso do Sul, Brazil. Habitat category: forest (for). The numbers following the underscore represent the scale of effect (in m).

**Model**	**Pseudo-R²**	**Slope**	**p**
Cabassous squamicaudis			
lm ($\delta^{13}C_c$ ~ for_500)	0.14	-0.27	0.10
lm ($\delta^{15}N_c$ ~ for_2000)	0.02	1.11	0.24

Table S12

Activity period, number of records, and overlap percentage among anteaters and armadillos in the Cerrado landscape, Mato Grosso do Sul, Brazil. Overlap values above 50% are highlighted in red.

**Species**	*C. squamicaudis*	*E. sexcinctus*	*M. tridactyla*	*P. maximus*	*D. novemcinctus*	*T. tetradactyla*
C. squamicaudis	-					
E. sexcinctus	0.63	-				
M. tridactyla	0.18	0.46	-			
P. maximus	0.04	0.17	0.58	-		
D. novemcinctus	0.02	0.18	0.59	0.83	-	
T. tetradactyla	0.21	0.27	0.61	0.76	0.64	-
**Activity**	Diurnal	Diurnal	Cathemeral	Nocturnal	Nocturnal	Nocturnal
**Records**	7	85	349	80	93	49

Assumptions check for linear regression models

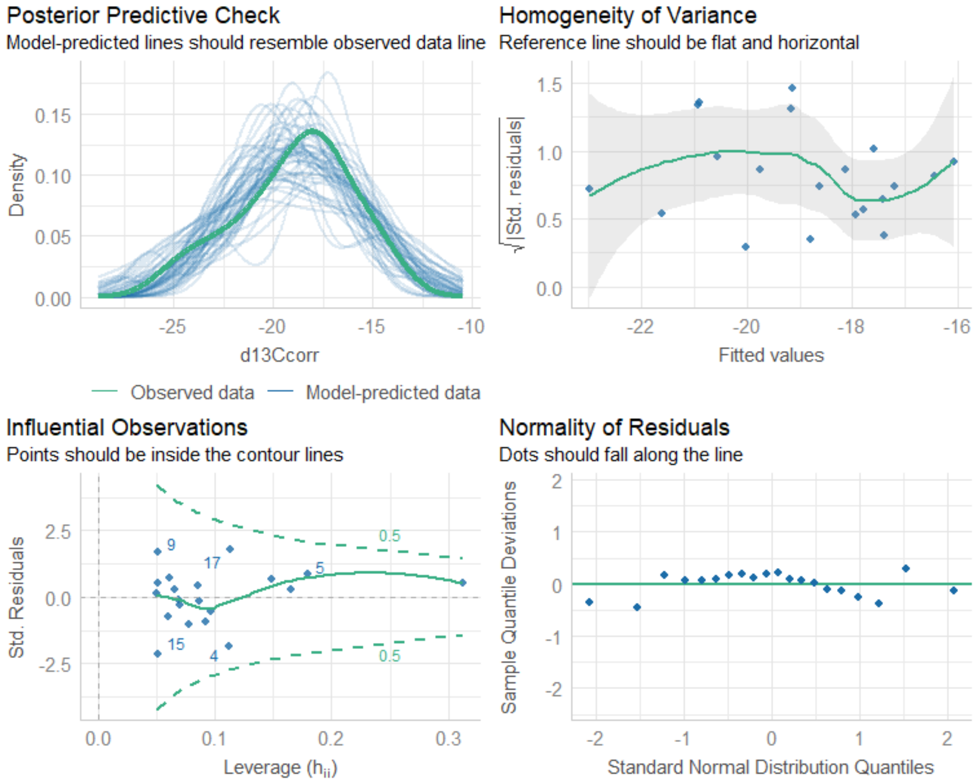

Figure S5

Dasypus novemcinctus: lm ($\delta^{13}C_c$ ~ mix_2000) – assumptions met; linear regression model employed

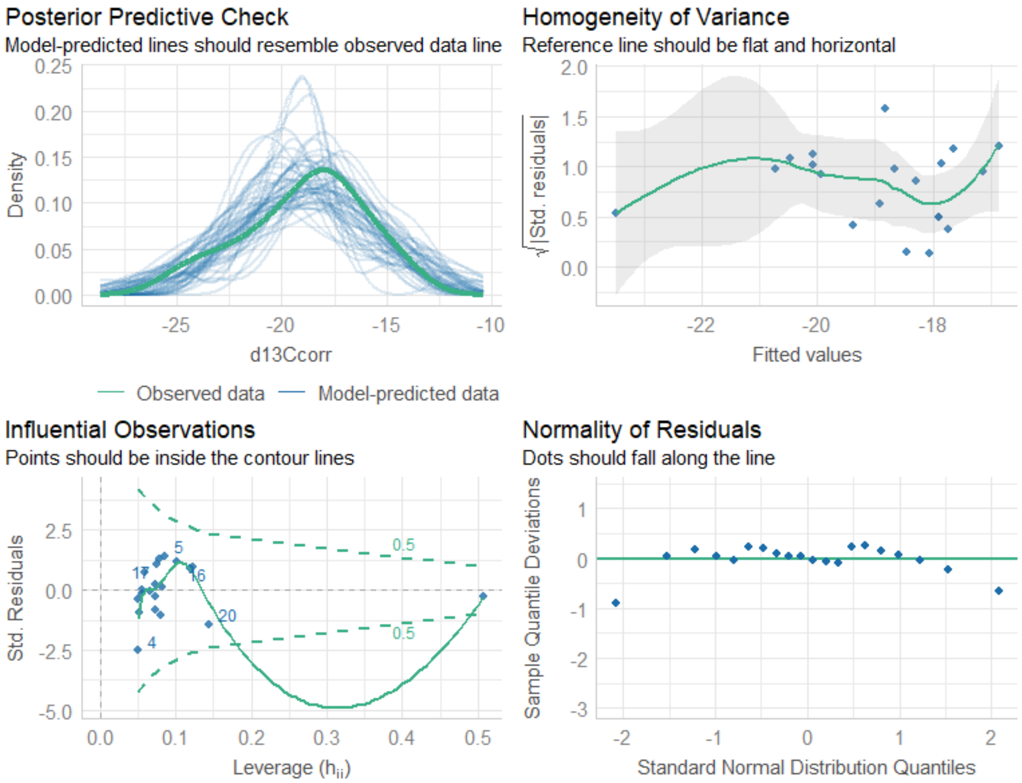

Figure S6

Dasypus novemcinctus: lm ($\delta^{13}C_c \sim$ for_2000) – assumptions met; linear regression model employed

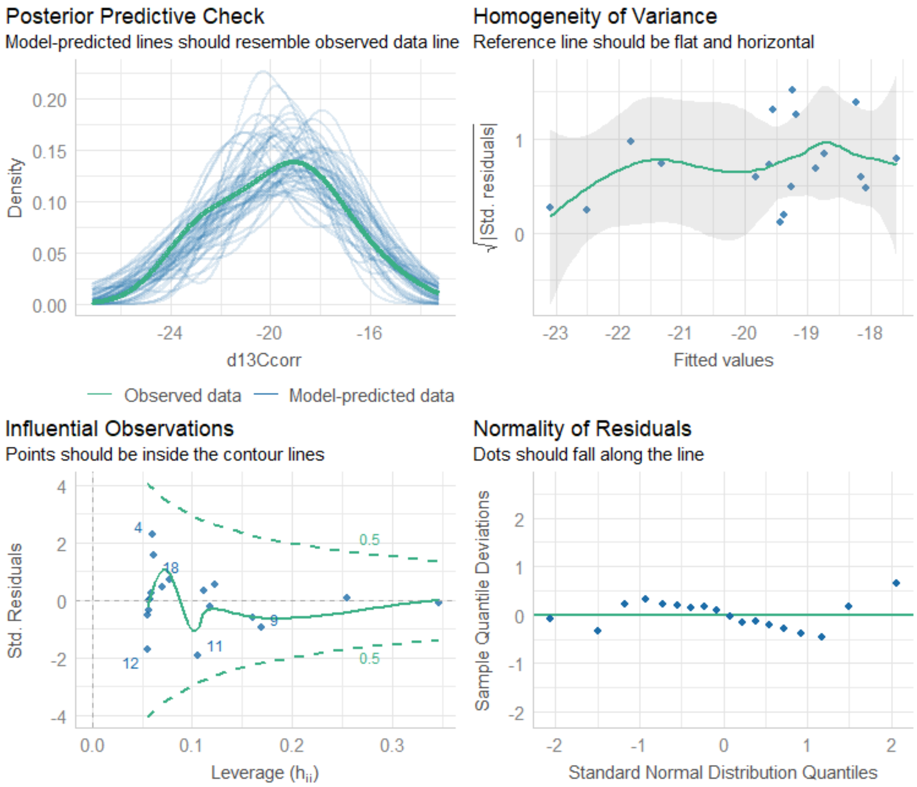

Figure S7

Priodontes maximus: lm ($\delta^{13}C_c \sim$ for_hr) – assumptions met; linear regression model employed

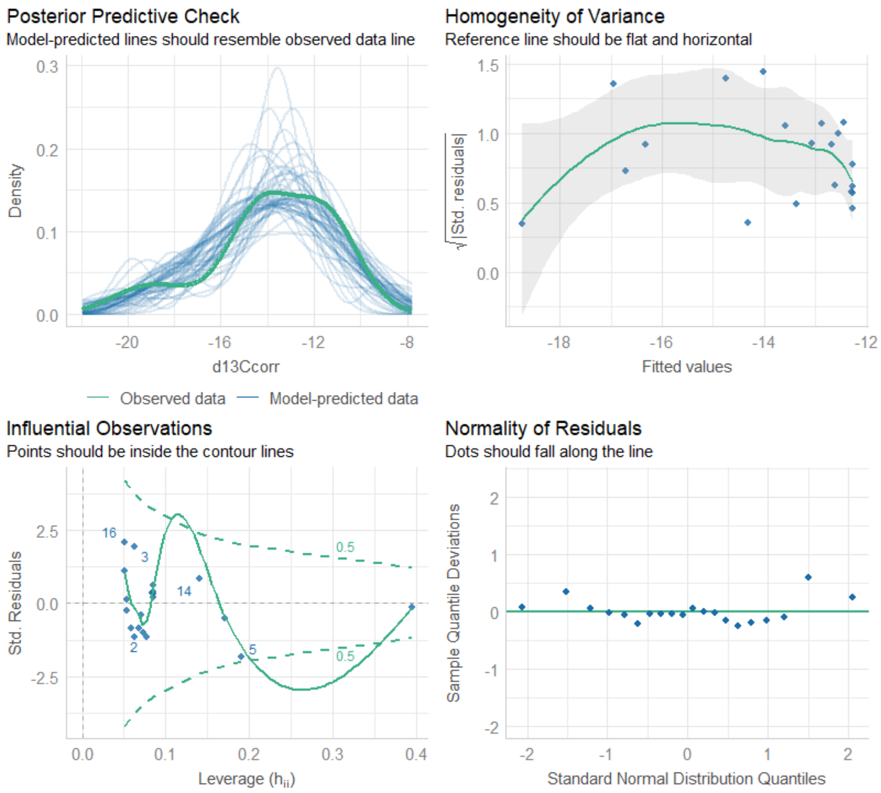

Figure S8

Myrmecophaga tridactyla: lm ($\delta^{13}C_c$ ~ sav_2000) – assumptions met; linear regression model employed

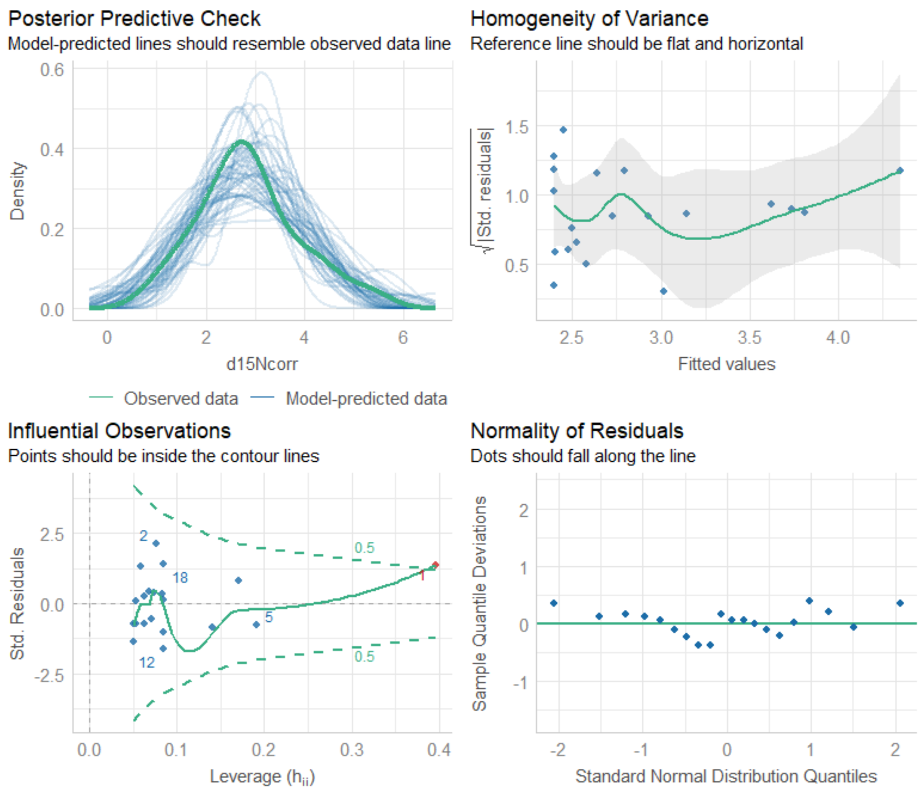

Figure S9

Myrmecophaga tridactyla: lm ($\delta^{15}N_c$ ~ for_1000) – influential outlier assumption not met; quadratic transformation of predictor was employed

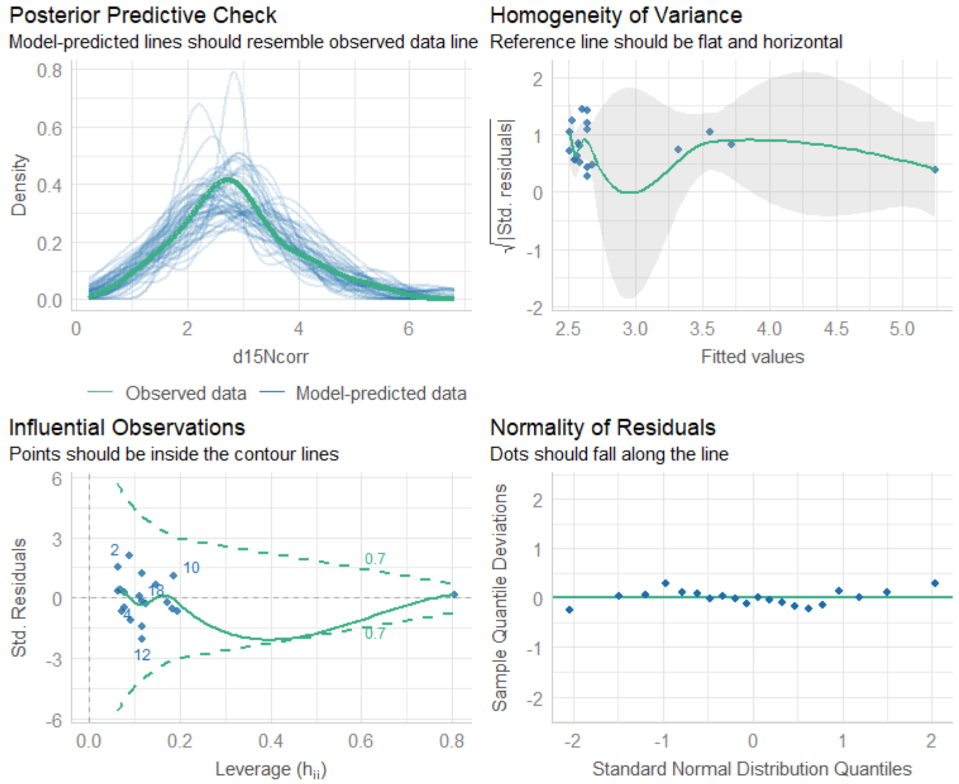

Figure S10

Myrmecophaga tridactyla: lm ($\delta^{15}\mathrm{N}_c \sim$ for_1000^2) – assumptions met; linear regression with quadratic transformation

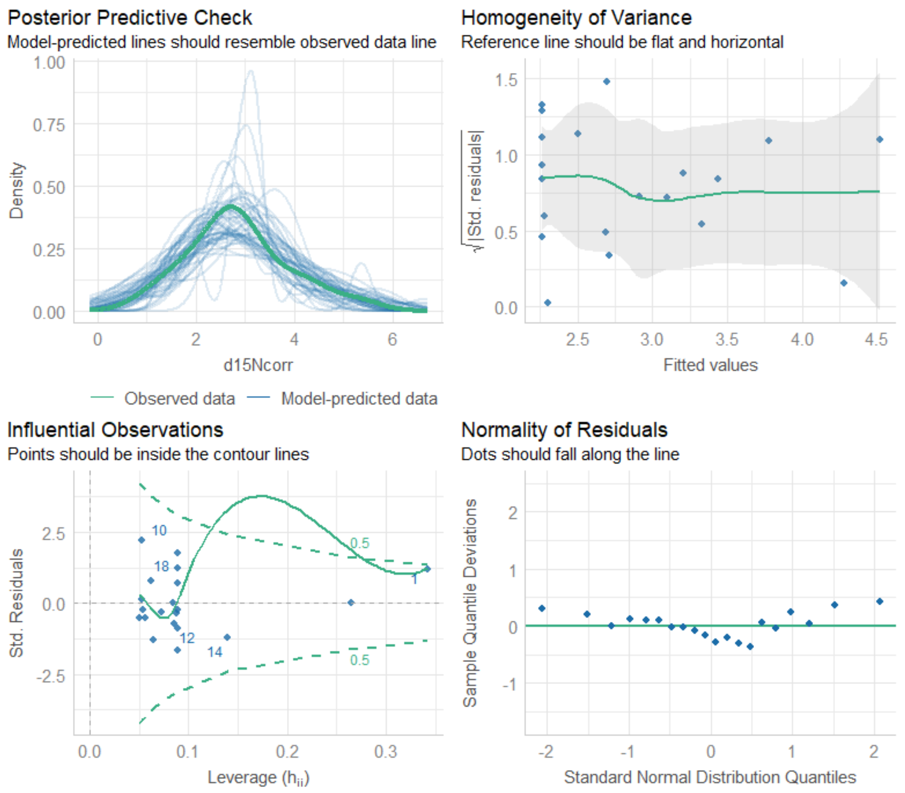

Figure S11

Myrmecophaga tridactyla: lm ($\delta^{15}\mathrm{N}_c \sim$ sav_2000) – assumptions met; linear regression model employed

Supporting references

Anacleto, T.C.D.S. (2007). Food habits of four armadillo species in the Cerrado area, Mato Grosso, Brazil. *Zoological Studies*, 46, 529–537.

Anacleto, T.C.S. & Marinho-Filho, J. (2001). *Hábito alimentar do tatu-canastra (Xenarthra, Dasypodidae) em uma área de cerrado do Brasil Central*. *Revista Brasileira de Zoologia*. scielo.

Anthony-Mathews. (1977). *Studies on termites from the Mato Grosso State, Brazil*. Academia Brasileira de Ciencias.

Araújo, T.G. de. (2013). Effect of air temperature on movement and activity patterns of southern tamanduas (*Tamandua tetradactyla*, Linnaeus, 1758). Dissertação de mestrado. Universidade Federal do Mato Grosso do Sul.

Barthe, M., Rancilhac, L., Arteaga, M.C., Feijó, A., Tilak, M.-K., Justy, F., *et al.* (2024). Exon capture museomics deciphers the nine-banded armadillo species complex and identifies a new species endemic to the Guiana Shield. *Systematic Biology*, syae027.

Bonato, V. (2002). Ecologia e história natural de tatus do cerrado de Itirapina, São Paulo (Xenarthra, Dasypodidae). Dissertação de mestrado. Universidade Estadual de Campinas.

Braga, F.G., Souza, N.J., Batista, A.C. & Lima, P.P. dos S. (2014). Consumo de Formigas Cortadeiras por Tamanduá-Bandeira Myrmecophaga tridactyla (Linnaeus, 1758) em Plantios de Pinus spp. no Paraná, Brasil. *Edentata*, 15, 1–8.

Carter, T.S., Superina, M. & Leslie Jr., D.M. (2016). Priodontes maximus (Cingulata: Chlamyphoridae). *Mammalian Species*, 48, 21–34.

Chatellenaz, M.L. & Mestres, J. (2023). The yellow armadillo as a potential predator of medium-sized vertebrates. *Food Webs*, 37, e00321.

Crawford, K., McDonald, R.A. & Bearhop, S. (2008). Application of stable isotope techniques to the ecology of mammals. *Mammalian Reviews*, 38, 87–107.

Dalponte, J.C. & Tavares-Filho, J.A. (2004). Diet of the Yellow Armadillo, Euphractus sexcinctus, in South-Central Brazil. *Edentata*, 2004, 37–41.

Deloss, A.X.R., Dröse, W., Rocha, M.M., Peters, F.B. & Kasper, C.B. (2024). Feeding habits of the lesser anteater Tamandua tetradactyla (Pilosa: Myrmecophagidae) in the Brazilian Pampa. *Studies on Neotropical Fauna and Environment*, 59, 457–463.

Desbiez, A.L.J., Kluyber, D., Massocato, G.F., Oliveira-Santos, L.G.R. & Attias, N. (2020). Spatial ecology of the giant armadillo Priodontes maximus in Midwestern Brazil. *Journal of Mammalogy*, 101, 151–163.

Desbiez, A.L.J., Massocato, G.F., Kluyber, D. & Santos, R.C.F. (2018). Unraveling the cryptic life of the southern naked-tailed armadillo, Cabassous unicinctus squamicaudis (Lund, 1845), in a Neotropical wetland: Home range, activity pattern, burrow use and reproductive behaviour. *Mammalian Biology*, 91, 95–103.

Drumond, M.A. (1992). Padrões de forrageamento do tamanduá-bandeira (*Myrmecophaga tridactyla*) no Parque Nacional da Serra da Canastra: Dieta, comportamento alimentar e efeito das queimadas. Dissertação de mestrado. Universidade Federal de Minas Gerais.

Feijó, A. & Anacleto, T.C. (2021). Taxonomic revision of the genus *Cabassous* McMurtrie, 1831 (Cingulata: Chlamyphoridae), with revalidation of *Cabassous squamicaudis* (Lund, 1845). *Zootaxa*, 4974, 47–78.

Ferreira, A.C., Caldato, N., Filho, W.R. & Iede, E.T. (2007). Composição da dieta de tamanduá-mirim (*Tamandua tetradactyla*). Presented at the VI Evento de Iniciação Científica da Embrapa Florestas.

Fuster, A., Diodato, L. & Contreras, J.H. (2018). Dieta de Myrmecophaga tridactyla (Pilosa: Myrmecophagidae) en pastizales y bosques del noreste de Santiago del Estero y su relación con las hormigas. *AZL*, 62, 1–9.

Gallo, J.A., Abba, A.M., Elizalde, L., Nucci, D.D., Ríos, T.A. & Ezquiaga, M.C. (2017). First study on food habits of anteaters, Myrmecophaga tridactyla and Tamandua tetradactyla, at the southern limit of their distribution. *Mammalia*, 81, 601–604.

Gaudin, T.J., Hicks, P. & Di Blanco, Y. (2018). Myrmecophaga tridactyla (Pilosa: Myrmecophagidae). *Mammalian Species*, 50, 1–13.

Gelman, A. & Shirley, K. (2011). Inference from simulations and monitoring convergence. In: *Handbook of Markov Chain Monte Carlo* (eds. Brooks, S., Gelman, A., Jones, G.L. & Meng, X.-. L.). Chapman and Hall/CRC, Boca Raton, pp. 163–174.

Gibb, G.C., Condamine, F.L., Kuch, M., Enk, J., Moraes-Barros, N., Superina, M., *et al.* (2016). Shotgun mitogenomics provides a reference phylogenetic framework and timescale for living xenarthrans. *Molecular Biology and Evolution*, 33, 621–642.

Hayssen, V. (2011). Tamandua tetradactyla (Pilosa: Myrmecophagidae). *Mammalian Species*, 43, 64–74.

IUCN - International Union for Conservation of Nature and Natural Resources. (2024). *The IUCN Red List of Threatened Species. Version 2024-1*. Available at: https://www.iucnredlist.org/. Last accessed .

Jiménez, N.L., Blanco, Y.E.D. & Calcaterra, L.A. (2018). Ant diversity in the diet of giant anteaters, *Myrmecophaga tridactyla* (Pilosa: Myrmecophagidae), in the Iberá Nature Reserve, Argentina. *Mastozoología Neotropical*, 25, 305–318.

Kéry, M. & Schaub, M. (2012). *Bayesian population analysis using WinBUGS*. Academic Press.

Loughry, W.J. & McDonough, C.M. (2013). *The nine-banded armadillo: a natural history*. University of Oklahoma Press.

Loughry, W.J., Superina, M., McDonough, C.M. & Abba, A.M. (2015). Research on armadillos: a review and prospectus. *Journal of Mammalogy*, 96, 635–644.

Lüdecke, D., Ben-Shachar, M.S., Patil, I., Waggoner, P. & Makowski, D. (2021). performance: an R package for assessment, comparison and testing of statistical models. *Journal of Open Source Software*, 6, 3139.

MacKenzie, D.I., Nichols, J.D., Royle, J.A., Pollock, K.H., Bailey, L.L. & Hines, J.E. (Eds.). (2017). *Occupancy Estimation and Modeling*. 2º edition. Academic Press, Boston.

Magioli, M., Attias, N., Massocato, G., Kluyber, D., Moreira, M.Z., Ferraz, K.M.P.M. de B., *et al.* (2023). What a few hairs can tell us about the resource use of giant armadillos. *Integrative Zoology*, 18, 129–142.

Magioli, M., Moreira, M.Z., Ferraz, K.M.B., Miotto, R.A., de Camargo, P.B., Rodrigues, M.G., *et al.* (2014). Stable isotope evidence of *Puma concolor* (Felidae) feeding patterns in agricultural landscapes in southeastern Brazil. *Biotropica*, 46, 451–460.

Magioli, M., Moreira, M.Z., Fonseca, R.C.B., Ribeiro, M.C., Rodrigues, M.G. & Ferraz, K.M.P.M.B. (2019). Human-modified landscapes alter mammal resource and habitat use and trophic structure. *Proceedings of the National Academy of Sciences*, 116, 18466–18472.

Martinelli, L.A., Nardoto, G.B., Soltangheisi, A., Reis, C.R.G., Abdalla-Filho, A.L., Camargo, P.B., *et al.* (2021). Determining ecosystem functioning in Brazilian biomes through foliar carbon and nitrogen concentrations and stable isotope ratios. *Biogeochemistry*, 154, 405–423.

McBee, K. & Baker, R.J. (1982). Dasypus novemcinctus. *Mammalian Species*, 1–9.

Medri, Í.M. (2008). Ecologia e história natural do tatu-peba, *Euphractus sexcinctus* (Linnaeus, 1758), no Pantanal da Nhecolândia, Mato Grosso do Sul. Tese de doutorado. Universidade de Brasília.

Medri, I.M., Mourão, G. de M. & Harada, A.Y. (2003). Dieta de tamanduá-bandeira (*Myrmecophaga tridactyla*) no Pantanal da Nhecolândia, Brasil. *Edentata*, 5, 29–34.

MMA – Ministério do Meio Ambiente. (2022). Portaria MMA nº 148, de 7 de junho de 2022: Altera os Anexos da Portaria nº 443, de 17 de dezembro de 2014, da Portaria nº 444, de 17 de dezembro de 2014, e da Portaria nº 445, de 17 de dezembro de 2014, referentes à atualização da Lista Nacional de Espécies Ameaçadas de Extinção. *Diário Oficial da União*, 108, 74.

Montgomery, G. (1985). Movements, foraging and food habits of the four extant species of neotropical vermilinguas (Mammalia; Myrmecophagidae). In: *The Evolution and ecology of armadillos, sloths, and vermilinguas*. Smithsonian Institution Press, pp. 365–377.

Nascimento, N.T., Attias, N., Galvão Santana, T., Rocha, M., Tibcherani, M., Massocato, G., *et al.* (2024). Dietary habits of the giant armadillo (Priodontes maximus) in the Brazilian wetlands. *Mammal Research*, 69, 423–434.

Noonan, M.J., Ascensão, F., Yogui, D.R. & Desbiez, A.L.J. (2022). Roads as ecological traps for giant anteaters. *Animal Conservation*, 25, 182–194.

Pasa, J.B., Hegel, C.G.Z. & Zanella, N. (2020). What are you eating? Stomach contents of roadkilled mammals of northern Rio Grande do Sul. *Oecologia Australis*, 24, 704–713.

Pitman, R.L., Powell, G., Cruz, D., Escobedo, M., Escobar, K., Vilca, V., *et al.* (2004). Habitat use and activity of the giant armadillo (*Priodontes maximus*): Preliminary data from southeastern Peru. Presented at the Annual Meeting of the Society for Conservation Biology, New York.

Post, D.M. (2002). Using stable isotopes to estimate trophic position: models, methods, and assumptions. *Ecology*, 83, 703–718.

Projeto MapBiomas. (2023). *Coleção 8 da série anual de mapas de cobertura e uso do solo do Brasil*. Available at: https://brasil.mapbiomas.org/. Last accessed 15 December 2023.

Redford, K.H. (1985). Food habits of armadillos (Xenarthra: Dasypodidae). In: *The evolution and ecology of armadillos, sloths and vermilinguas* (ed. Montgomery, G.G.). Smithsonian Institution Press, Washington, D.C., pp. 429–437.

Redford, K.H. (1986). Dietary specialization and variation in two mammalian myrmecophages (variation in mammalian myrmecophagy). *Revista Chilena de Historia Natural*, 59, 201–208.

Redford, K.H. & Wetzel, R.M. (1985). Euphractus sexcinctus. *Mammalian Species*, 1–4.

Rodrigues, T.F. & Chiarello, A.G. (2018). Native forests within and outside protected areas are key for nine-banded armadillo (*Dasypus novemcinctus*) occupancy in agricultural landscapes. *Agriculture, Ecosystems & Environment*, 266, 133–141.

Royle, J.A. & Dorazio, R.M. (Eds.). (2008). *Hierarchical Modeling and Inference in Ecology*. Academic Press, San Diego.

Sandoval-Gómez, V.E., Ramérez-Chaves, H.E. & Marín, D. (2012). Registros de Hormigas Y Termitas Presentes en la Dieta de Osos Hormigueros (Mammalia: Myrmecophagidae) en Tres Localidades de Colombia. *Edentata*, 13, 1–9.

Schaller, G.B. (1983). Mammals and their biomass on a Brazilian ranch. *Arq. Zool.*, 31, 1–36.

Shaw, J.H., Carter, T.S. & Machado-Neto, J.C. (1985). Ecology of the giant anteater *Myrmecophaga tridactyla* in Serra da Canastra, Minas Gerais, Brazil: a pilot study. In: *The Evolution and ecology of armadillos, sloths, and vermilinguas*. Smithsonian Institution Press, pp. 379–384.

Silva, M.M. (2019). Análise da dieta de *Tamandua tetradactyla* Linnaeus, 1758 (Pilosa: Myrmecophagidae) do Rio de Janeiro e Minas Gerais, Brasil. Dissertação de mestrado. Universidade Federal Rural do Rio de Janeiro.

Silva, T.S.G. da, Attias, N., Nascimento, N.T., Tibcherani, M. & Desbiez, A.J.L. (2023). The diet of the giant anteater, *Myrmecophaga tridactyla* Linnaeus 1758, in the Cerrado of Mato Grosso do Sul, Brazil.

Sousa, E.A. & Messias, M.R. (2006). Dieta de Fêmeas de *Tamandua tetradactyla* (Pilosa: Myrmecophagidae) ocorrentes nas áreas de impacto direto da usina hidrelétrica Santo Antônio – Porto Velho/RO. Presented at the 6º Congresso Brasileiro de Mastozoologia, Corumbá.

Vaz, V.C., Santori, R.T., Jansen, A.M., Delciellos, A.C. & D'Andrea, P.S. (2012). Notes on Food Habits of Armadillos (Cingulata, Dasypodidae) and Anteaters (Pilosa, Myrmecophagidae) at Serra Da Capivara National Park (PiauÍ State, Brazil). *Edentata*, 13, 84–89.

Wetzel, R.M. (1985a). Taxonomy and distribution of armadillos, Dasypodidae. In: *The evolution and ecology of armadillos, sloths and vermilinguas*. Smithsonian Institution Press, pp. 23–46.

Wetzel, R.M. (1985b). The identification and distribution of recent Xenarthra (= Edentata). In: *The evolution and ecology of armadillos, sloths, and vermilinguas*. Smithsonian Institution Press, pp. 5–21.

Yone, S., Bueno, C., Costa, J.P., Santos, L.A.O. & Olifiers, N. (2020). Diet of *Dasypus novemcinctus* Linnaeus, 1758 in Atlantic Forest area of southeast of Brazil. Presented at the Congresso Internacional de Conservação de Xenarthra.

Zambrini, A.C.V. (2015). Ecologia alimenar de tatu-peba, *Euphractus sexcinctus* (Linnaeus, 1758), na fazenda Nhumirim, Pantanal da Nhecolândia, MS. Trabalho de Conclusão de Curso. Universidade Estadual Paulista "Júlio de Mesquita Filho."
